# Supplementary material for: Health-seeking behaviours in a malaria endemic district in Lao People’s Democratic Republic: a mixed methods study
Source: BMJ Open. 2021 Dec 13;11(12):e055350. doi: 10.1136/bmjopen-2021-055350 (PMC8671991; doi:10.1136/bmjopen-2021-055350)
Supplement: Supplementary data [file bmjopen-2021-055350supp003.pdf]

Supplementary 3 Characteristics of the villagers in the focus group discussions

No.	Village	Content	ID	Unique ID	Participants
1	A	Male FGD	5.59	BM1.1	60s, Retiree
			2.67	BM1.2	40s, Teacher
			2.112	BM1.3	40s, Government servant
			4.113	BM1.4	20s, Soldier
2	A	Male FGD	6.69	BM2.1	50s, Farmer
			5.126	BM2.2	20s, Farmer
			5.129	BM2.3	50s, Farmer
3	A	Female FGD	5.6	BF1.1	30s, Businesswoman
			6.50	BF1.2	50s, Farmer
			2.102	BF1.3	40s, Farmer
4	A	Female FGD	1.122	BF2.1	30s, Farmer
			4.139	BF2.2	40s, Farmer
			3.140	BF2.3	40s, Government servant
5	B	Male FGD	3.61	NM1.1	20s, Government servant
			3.76	NM1.2	30s, Farmer
			4.77	NM1.3	20s, Farmer
6	B	Male FGD	4.87	NM2.1	30s, Farmer
			3.89	NM2.2	30s, Farmer
			1.91	NM2.3	10s, Farmer
7	B	Male FGD	2.93	NM3.1	40s, Farmer
			1.95	NM3.2	30s, Farmer
			1.102	NM3.3	20s, Farmer
8	B	Female FGD	5.41	NF1.1	40s, Farmer
			1.54	NF1.2	40s, Farmer
			2.55	NF1.3	40s, Farmer
9	B	Female FGD	1.98	NF2.1	20s, Farmer
			2.101	NF2.2	20s, Farmer
			3.106	NF2.3	30s, Farmer
10	B	Female FGD	3.99	NF3.1	20s, Farmer
			4.100	NF3.2	40s, Farmer
			2.104	NF3.3	20s, Farmer
