# Supplementary material for: Health-seeking behaviours in a malaria endemic district in Lao People’s Democratic Republic: a mixed methods study
Source: BMJ Open. 2021 Dec 13;11(12):e055350. doi: 10.1136/bmjopen-2021-055350 (PMC8671991; doi:10.1136/bmjopen-2021-055350)
Supplement: Supplementary data [file bmjopen-2021-055350supp004.pdf]

Supplementary 4 Characteristics of the healthcare workers in the in-depth interviews

No.	Village	Content	Unique ID	Age	Sex
1	A	Healthcare worker IDI	BH1	30s	Female
2	A	Healthcare worker IDI	BH2	40s	Female
3	A	Village Health Volunteer IDI	BV1	50s	Male
4	B	Healthcare worker IDI	NH1	30s	Male
5	B	Healthcare worker IDI	NH2	20s	Female
6	B	Healthcare worker IDI	NH3	30s	Female
7	B	Village Health Volunteer IDI	NV1	50s	Male

Ong KIC, *et al. BMJ Open* 2021; 11:e055350. doi: 10.1136/bmjopen-2021-055350
