# Supplementary material for: Health-seeking behaviours in a malaria endemic district in Lao People’s Democratic Republic: a mixed methods study
Source: BMJ Open. 2021 Dec 13;11(12):e055350. doi: 10.1136/bmjopen-2021-055350 (PMC8671991; doi:10.1136/bmjopen-2021-055350)
Supplement: Supplementary data [file bmjopen-2021-055350supp005.pdf]

Supplementary 5 Themes from the focus group discussions of the villagers

Themes	Quotes, Unique ID
Attributing the cause of malaria to something else other than being bitten by mosquitoes	1. Because your house is not clean. If you drink water with mosquito eggs, you will get fever. *BF 2.3 40s, Government servant*<br><br>2. When I had malaria, they used '*pee pop*' (ghost) to exorcize it away. *BF 2.1 30s, Farmer*<br><br>3. Drinking unclean water. *BM1.4 20s, Soldier*<br><br>4. Drinking unclean water, you will get malaria. *NM 2.3 10s, Farmer*<br><br>5. Scared of malaria because scared of '*pop hed*' (caused by ghost). *NF1.2 40s, Farmer*
Attributing the cause of malaria to being bitten by mosquitoes	1. Mosquito bite, not sleeping under the bed net, not taking care of oneself. Not protecting oneself. *BF1.1 30s, Businesswoman*<br><br>2. Caused by mosquitoes. You must be bitten by mosquitoes first. *BF 2.1 30s, Farmer*<br><br>3. Because of mosquito bites. When you do not sleep under the bed net, the mosquitoes will bite you. *NM1.1 20s, Government servant*<br><br>4. Do not sleep under the bed net, being bitten by mosquitoes. *NM3.2 30s, Farmer*
Describing an illness using local terms	1. '*Kai luad niao*' (sticky blood fever), when you are dehydrated, your blood will be sticky. When you drink water, your blood will dilute. *BF1.3 40s, Farmer*<br><br>2. Among men, '*kai luad niao*' (sticky blood fever) is also very common. There were two cases this year, and they died. *BM1.4 20s, Soldier*<br><br>3. '*Kai nyoong*' (literally translated from Lao as 'mosquito fever', usually referred to malaria) in Katang language is called '*ae muay*'. *NF1.1 40s, Farmer*
Expressing hope to	1. I just hope the health center will be well-equipped with medicines

Ong KIC, *et al. BMJ Open* 2021; 11:e055350. doi: 10.1136/bmjopen-2021-055350

improve the health center	and such, and I hope things will get better. I am not criticizing them, I understand that there is not enough budget. *BF1.3 40s, Farmer*<br><br>2. I hope the health center will be better equipped, with Echo machine, with qualified medical doctors, surgical services. *NM1.2 30s, Farmer*<br><br>3. I hope the staff will be more knowledgeable on the treatments. *NM3.2 30s, Farmer*<br><br>4. Improve the health center, I hope they have X-ray machines, proper equipment. *NF1.1 40s, Farmer*<br><br>5. I hope there will be more medical personnel from other villages stationed here and live here so it will be convenient for us to call on them. *NF1.1 40s, Farmer*<br><br>6. I hope the health center will be cleaner. There are mosquitoes here at the health center too, so you might even get malaria here! *NF2.2 20s, Farmer*<br><br>*7.* I hope the staff at the health center will further their studies and improve their knowledge to help the villagers become healthy. *NM3.1, 40s Farmer*
Expressing hope to improve the district hospital	1. I hope there will also be a new building at the district hospital. Sometimes there are not enough beds for the patients and the family members have no place to stay. *NM1.1 20s, Government servant; NM1.2 30s, Farmer; NM1.3 20s, Farmer*
Recalling personal experience	1. We do not have oxygen here in the district hospital. I gave birth to a premature baby once and it died because we did not have oxygen. We were going to the Savannakhet provincial hospital and the baby died on the way in Pakxong. No incubator too. *BF1.1 30s, Businesswoman*<br><br>2. When I got fever and the health staff gave me the wrong medicine. They do not know. *NM3.3 20s, Farmer*
Expressing opinion	1. Overall everything is good but only one thing is that medicine is

on the health center	always not enough. *BM1.2 40s, Teacher*<br><br>2. Sometimes, when people are shabbily dressed, the staff do not want to treat them. *BM1.4 20s, Soldier*<br><br>3. If you pay less you get only 'paracetamol'. You won't get well. Last time I had to pay but I felt I recovered fast. Now I pay only 5000 kip I don't get well soon. *NF2.1 20s, Farmer; NF2.2 20s, Farmer; NF2.3 30s, Farmer*
Feeling towards malaria	1. Mosquitoes are scarier than tigers. *NM1.2 30s, Farmer*<br><br>2. Scary! Mosquitoes are scarier than tigers. *NM3.1 40s, Farmer*
Expressing opinion towards malaria elimination	1. Yes, malaria can be eliminated. Destroy the mosquitoes. Spray them. They will be gone. *BF1.3 40s, Farmer*<br><br>2. Elimination is difficult because when people go to the forest, they drink water from the stream. *BF 2.3 40s, Government servant*<br><br>3. It's difficult to eliminate malaria. The only way is to treat. *BM1.4 20s, Soldier*<br><br>4. Cannot be eliminated because our people depend on the forest to make a living, they will just sleep when it gets dark when they are in the forest. *BM2.3 50s, Farmer*<br><br>5. Yes, take care of hygiene, protect oneself when going to the forest. *BM1.4 20s, Soldier*
Expressing difficulties in getting health services	1. '*Kon luai bor dai kau kuk, kon tuk bor dai kau hong mor*' (The rich do not go to jail, the poor do not go to the hospital). *NM1.3 20s, Farmer*<br><br>2. I hope the roads will also be improved. When it is raining, people die because they are not sent to the hospital in time. *NM1.2 30s, Farmer*
Mentioning about the National Health Insurance ("*Kor Por Sor*")	1. I will go to the health center first, because I have to pay only 5000 kip. If I go to a private clinic, I will have to pay at least 50000 kip. *BF 2.1 30s, Farmer*
