# Supplementary material for: Health-seeking behaviours in a malaria endemic district in Lao People’s Democratic Republic: a mixed methods study
Source: BMJ Open. 2021 Dec 13;11(12):e055350. doi: 10.1136/bmjopen-2021-055350 (PMC8671991; doi:10.1136/bmjopen-2021-055350)
Supplement: Supplementary data [file bmjopen-2021-055350supp006.pdf]

Supplementary 6 Themes from the in-depth interviews of the healthcare workers

Themes	Quotes, Unique ID
Mentioning about the National Health Insurance ("*Kor Por Sor*")	1. Now there is the *'Kor Por Sor'* (National Health Insurance) system, where you have to pay only 5000 kip, they will come even when they have common cold. *BH1, 30s Female*<br>2. Because of *'Kor Por Sor'* (National Health Insurance), more people come to seek treatment at this health center. *NH1, 30s Male*<br>3. Because of *'Kor Por Sor'* (National Health Insurance), we have more patients who come here for treatment. *NH2, 30s Female*
Expressing opinion towards malaria elimination	1. Yes, you can eliminate malaria. You should sleep under the long-lasting insecticide-treated bed nets. In addition, we should also train the village health volunteers to use the rapid diagnostic test (RDT) so we can eliminate malaria. *BH1, 30s Female*<br>2. It's difficult to eliminate because people here make a living by going to the mountains, to the forests. We can't stop them from going. They need to make a living. *NH2, 30s Female*<br>3. You can't eliminate 100%. People here work in the field, go to the forest, go to the mountains. *NH3, 20s Female*
Describing an illness using local terms	1. I heard the villagers mentioned '*luad niao*', '*luad niao*', is there such thing? *BH2, 40s Female*
Describing the villagers' health-seeking behavior	1. Yes, when they fall sick, they will go to the '*mor pee*' (witch doctor) first. But only for some families. Usually the old. *BV1, 50s Male*<br>2. The villagers come to the health center directly as we do not encourage them to go the village health volunteer first. I am afraid that the village health volunteer do not know the right way to treat the patients. *BH2, 40s Female*
Feeling towards the job	1. I am proud of my work. I am a nurse but I also function as a 'doctor' here. I have to work more than what I studied. It is very challenging sometimes but the longer I work, the more experience

	I have. *NH2, 30s Female*<br><br>2. I am satisfied with my work. Because this work can save lives. *NV1, 50s Male*
Expressing hope to improve the health center	1. The knowledge and capability. I want to learn more. Another thing is the building. Too small. When people are sick they can't sleep here. If possible, I hope for more advanced equipment too. *NH1, 30s Male*<br><br>2. I hope the health center will be expanded. I also want to have machines that can check blood or Echo machine. I also hope that a medical doctor will be stationed here. *NH2, 30s Female*<br><br>3. At this health center the manpower is not enough, we also do not have enough knowledge. I hope people with knowledge will come here to work at this health center. I also hope this health center will be expanded. It is very crowded when there are many sick people and people who give birth. *NH3, 20s Female*
Expressing hope to improve the living condition of the people	1. I hope there will be toilets in every house. *NV1, 50s Male*
