# Supplementary material for: Health-seeking behaviours in a malaria endemic district in Lao People’s Democratic Republic: a mixed methods study
Source: BMJ Open. 2021 Dec 13;11(12):e055350. doi: 10.1136/bmjopen-2021-055350 (PMC8671991; doi:10.1136/bmjopen-2021-055350)
Supplement: Author's manuscript [file bmjopen-2021-055350.draft_revisions.pdf]

BMJ Open is committed to open peer review. As part of this commitment we make the peer review history of every article we publish publicly available.

When an article is published we post the peer reviewers' comments and the authors' responses online. We also post the versions of the paper that were used during peer review. These are the versions that the peer review comments apply to.

The versions of the paper that follow are the versions that were submitted during the peer review process. They are not the versions of record or the final published versions. They should not be cited or distributed as the published version of this manuscript.

BMJ Open is an open access journal and the full, final, typeset and author-corrected version of record of the manuscript is available on our site with no access controls, subscription charges or pay-per-view fees (http://bmjopen.bmj.com).

If you have any questions on BMJ Open's open peer review process please email info.bmjopen@bmj.com

BMJ Open

Health-seeking behaviors in a malaria endemic district in Lao People's Democratic Republic: a mixed methods study

Journal:	*BMJ Open*
Manuscript ID	bmjopen-2021-055350
Article Type:	Original research
Date Submitted by the Author:	11-Jul-2021
Complete List of Authors:	Ong, Ken; The University of Tokyo, Department of Community and Global Health<br>Khattignavong, Phonepadith; Institut Pasteur du Laos<br>Keomalaphet, Sengdeuane; Institut Pasteur du Laos<br>Iwagami, Moritoshi; Institut Pasteur du Laos; National Center for Global Health and Medicine Research Institute, Department of Tropical Medicine and Malaria<br>Brey, Paul; Institut Pasteur du Laos<br>Kano, Shigeyuki; National Center for Global Health and Medicine Research Institute, Department of Tropical Medicine and Malaria<br>Jimba, Masamine; The University of Tokyo, Community and Global Health
Keywords:	Public health < INFECTIOUS DISEASES, PUBLIC HEALTH, QUALITATIVE RESEARCH

SCHOLARONE™
Manuscripts

I, the Submitting Author has the right to grant and does grant on behalf of all authors of the Work (as defined in the below author licence), an exclusive licence and/or a non-exclusive licence for contributions from authors who are: i) UK Crown employees; ii) where BMJ has agreed a CC-BY licence shall apply, and/or iii) in accordance with the terms applicable for US Federal Government officers or employees acting as part of their official duties; on a worldwide, perpetual, irrevocable, royalty-free basis to BMJ Publishing Group Ltd ("BMJ") its licensees and where the relevant Journal is co-owned by BMJ to the co-owners of the Journal, to publish the Work in this journal and any other BMJ products and to exploit all rights, as set out in our licence.

The Submitting Author accepts and understands that any supply made under these terms is made by BMJ to the Submitting Author unless you are acting as an employee on behalf of your employer or a postgraduate student of an affiliated institution which is paying any applicable article publishing charge ("APC") for Open Access articles. Where the Submitting Author wishes to make the Work available on an Open Access basis (and intends to pay the relevant APC), the terms of reuse of such Open Access shall be governed by a Creative Commons licence – details of these licences and which Creative Commons licence will apply to this Work are set out in our licence referred to above.

Other than as permitted in any relevant BMJ Author's Self Archiving Policies, I confirm this Work has not been accepted for publication elsewhere, is not being considered for publication elsewhere and does not duplicate material already published. I confirm all authors consent to publication of this Work and authorise the granting of this licence.

For peer review only

Title: Health-seeking behaviors in a malaria endemic district in Lao People's Democratic Republic: a mixed methods study

Ken Ing Cherng Ong[1,2*], Phonepadith Khattignavong[2,3], Sengdeuane Keomalaphet[2,3], Moritoshi Iwagami[2,3,4], Paul T. Brey[2,3], Shigeyuki Kano[2,3,4], Masamine Jimba[1,2]

[1]Department of Community and Global Health, Graduate School of Medicine, The University of Tokyo, Tokyo, Japan

[2]SATREPS project (JICA/AMED) for Parasitic Diseases, Vientiane, Lao PDR

[3]Institut Pasteur du Laos, Ministry of Health, Samsenthai Road, Ban Kao-Gnot, Sisattanak District, P.O. Box 3560, Vientiane, Lao PDR

[4]Department of Tropical Medicine and Malaria, Research Institute, National Centre for Global Health and Medicine, 1-21-1 Toyama, Shinjuku, Tokyo 162-8655, Japan

***Corresponding author: Ken Ing Cherng Ong (kenicong@m.u-tokyo.ac.jp)**

Word counts: 4265 words

For peer review only - http://bmjopen.bmj.com/site/about/guidelines.xhtml

Abstract

Objectives: As malaria continues to threaten lives, the effort to rid the world of malaria has regained momentum and an ambitious goal to eradicate malaria by 2050 has been set again. Social medicine approach is important and could synergize the biomedical approach towards malaria elimination. This mixed methods study was conducted to explore the barriers and facilitators for health-seeking behaviors in a malaria endemic district in Lao PDR.

Design: A convergent mixed methods design.

Setting: Two malaria endemic villages in Thapangthong district, Savannakhet Province, Lao PDR.

Participants: Villagers and healthcare workers in the two villages in Thapangthong district.

Methods: In the quantitative part, a pre-tested questionnaire was used to identify the health-seeking behaviors of the villagers. In the qualitative part, focus group discussions were employed to explore health-seeking behaviors of the villagers and in-depth interviews were used to explore the perceptions of the healthcare workers. Descriptive statistics were computed and multiple logistic regressions were used to identify the factors associated with perceived severity and perceived susceptibility. Thematic analysis was used to analyze the qualitative data. Joint displays were used to integrate the quantitative and qualitative results.

Results: In the quantitative part, data were collected from 313 villagers from both villages. Most villagers from both villages would seek treatment at the health center or the district

For peer review only - http://bmjopen.bmj.com/site/about/guidelines.xhtml

hospital. However, qualitative data revealed that villagers faced problems such as lack of medicines and medical equipment. Despite this, the villagers still preferred to seek treatment at the health center as the National Health Insurance was introduced.

Conclusions: In conclusion, public health facility usage was very high but barriers existed. Effective policy and enabling environment such as the introduction of the National Health Insurance could help accelerate the progress towards the malaria elimination goal. Moreover, the benefits could go beyond the context of malaria.

For peer review only

For peer review only - http://bmjopen.bmj.com/site/about/guidelines.xhtml

Strengths and limitations of this study

- This study used both quantitative and qualitative methods to explore the health-seeking behaviors in a malaria endemic district in Lao PDR.
- In addition, perspectives from both the villagers and the healthcare providers were obtained and analyzed.
- This provided a comprehensive picture of the health-seeking behaviors in the malaria endemic district.
- Generalizability might be an issue but the lessons learned might be transferable to other malaria endemic areas in Lao PDR and other similar settings.

For peer review only

For peer review only - http://bmjopen.bmj.com/site/about/guidelines.xhtml

Introduction

Malaria threatens lives [1]. In 2018; 405,000 lives were lost to malaria and 228 million cases were reported [2]. Moreover, 272,000 deaths (67%) occurred in children under 5 years of age [2]. Currently, the effort to rid the world of malaria has regained momentum and an ambitious goal to eradicate malaria by 2050 has been set once again [3]. However, the application of the same, presently available tools and approaches will not help us achieve the goal [4].

In the Greater Mekong Subregion (GMS), malaria mortality has substantially decreased in the past decade [5]. However, malaria elimination efforts in many GMS countries have been hampered by difficulties, such as large movements of people across borders, diverse geographical terrains, and the availability and access to counterfeit antimalarials [6]. Furthermore, these factors have also contributed to the emergence of antimalarial drug resistance [7].

In the Lao People's Democratic Republic (Lao PDR), which is situated in the GMS, the key risk populations have been identified as ethnic minority groups living in remote forested and mountainous areas, plantation workers, migrant workers, and the soldiers [8, 9]. Lao PDR is a linguistically and ethnically diverse nation [10]. Ethnic minorities in Lao PDR usually inhabit remote mountainous and forest areas and are usually characterized by extreme poverty [8]. These ethnic minorities usually have their own distinct languages and many do

For peer review only - http://bmjopen.bmj.com/site/about/guidelines.xhtml

not speak Lao [5, 8]. As a result, dissemination of health messages is challenging [8]. Moreover, many ethnic minorities are animists with their own unique beliefs towards illness [10, 11]. Consequently, many people from these groups still resort to traditional medicine and religious/animistic rituals for treatment [11].

The level of knowledge on modern medicine in Lao PDR is still low [12, 13]. To complicate matters further, substandard and counterfeit medicines are still available in private pharmacies [12, 14]. In the context of malaria, this could hamper the ongoing efforts of malaria elimination, endanger patients' lives, and worsen the drug resistance problem in Lao PDR [12, 14].

To achieve the Sustainable Development Goal 3: Good Health & Wellbeing for All; a key element must be attained: achieving Universal Health Coverage (UHC). To this end, the Lao Government has introduced the National Health Insurance (known by the locals as "*Kor Por Sor*") scheme in 2016. In this scheme, all Lao citizens can receive treatments at public health facilities at a very minimal cost. For example, for outpatient visits at a village health center, villagers pay only 5000 kip (approximately US$ 0.55) per visit. However, the implementation of the National Health Insurance scheme is inconsistent throughout the country and some villages only fully implemented this scheme two years after the official introduction by the Lao Government [15].

To accelerate the progress towards malaria elimination by 2030, understanding the

For peer review only - http://bmjopen.bmj.com/site/about/guidelines.xhtml

level of knowledge of the populations in malaria endemic areas on modern medicine and their health-seeking behaviors is critical to inform the policymakers on the types of interventions and treatment plans and policies to improve healthcare delivery in Lao PDR. The objective of this study was to explore health-seeking behaviors from the perspectives of the villagers and the frontline healthcare workers in a malaria endemic area in Lao PDR.

Methods

Study design and settings

A convergent mixed methods design was used where both quantitative and qualitative data were simultaneously collected and integrated [16]. In this study, quantitative data were collected from the villagers, and qualitative data from both the villagers and the healthcare workers.

This study was conducted in October 2018 in Thapangthong district, Savannakhet province, Lao PDR. This district was chosen because of the high number of reported malaria cases. In 2018, out of 2245 reported malaria positive cases in Savannakhet province, 323 were from Thapangthong district [Data obtained by personal communication with the Center of Malariology, Parasitology and Entomology, Lao PDR]. Two villages (Villages A and B) were chosen after consultation with the District Health Office in Thapangthong district based on the number of highest malaria cases and consultation with the village headmen.

Thapangthong district comprises of 42 villages, and the population as of October

For peer review only - http://bmjopen.bmj.com/site/about/guidelines.xhtml

2018 was 42,563, of which 21,157 were female villagers. The district has one district hospital and the ethnic composition of this district is 42% Lao ethnic group and 58% Katang ethnic group. The population of Village A was 1149 villagers living in 215 households. Out of 1149 villagers, 570 were women. The major ethnic group in Village A is Lao. Village B has 753 villagers living in 125 households. Out of 753 villagers, 324 were women. The major ethnic group in Village B is Katang.

Study participants and recruitment

Study participants in this study were villagers and healthcare workers aged 18 years and above living and working in the two villages. After Village A and Village B were chosen, the village headmen of each village were informed the purpose of this study. For quantitative data collection, the village headmen informed the whole village a few days before the study took place, and requested a representative of each household who agreed and were willing to participate in the study to come at the specified time and place.

For qualitative data collection, villagers who finished the quantitative interview were personally approached and informed about the focus group discussions (FGDs). All the villagers who volunteered were included. For the in-depth interviews (IDIs) of the healthcare workers, all the healthcare workers were approached and informed of the purpose of the study. All those who agreed to participate were interviewed.

Quantitative part

For peer review only - http://bmjopen.bmj.com/site/about/guidelines.xhtml

A structured questionnaire was created by adapting the content from the latest Lao Social Indicator Survey [17], Malaria Indicator Survey from Roll Back Malaria [18, 19], and clinical vignettes from a study by Mebratie et al [20]. The questionnaire consisted of seven sections: health-seeking behaviors, knowledge on malaria and modern medicine, perceived severity and perceived susceptibility, perceived benefits, self-efficacy, clinical vignettes, and socioeconomic characteristics.

The questionnaires were translated into Lao by two authors (P.K. and S. Ke., both medical doctors and malaria researchers) who are Lao native speakers and fluent in English. They also checked each other's translation for accuracy and checked the questionnaire for content and face validity. Before data collection, six research assistants were trained on the content of the questionnaire and ethical issues such as privacy protection of the participants. The questionnaire was pre-tested in another village in the same district among 14 villagers. After the pretest, a meeting with all the research assistants was held to revise the questionnaires for easier understanding based on their feedbacks.

Six research assistants collected the quantitative data by interviewer-administered questionnaire using KoBo Collect application downloaded on password protected Android tablets provided to each research assistant. Each villager who finished answering the questionnaire was provided with a bag of sanitary items such as soap and washing detergents as an incentive.

For peer review only

Descriptive statistics were computed to summarize the socio-demographic and socio-economic characteristics of the participants. Chi-square test was used for categorical variables. Multiple logistic regressions were used to identify the factors associated with perceived severity and perceived susceptibility. For clinical vignettes, providers such as village health volunteers, health centers, district hospitals, and provincial hospitals were categorized as ‘public health facilities’. Other providers were grouped as ‘others’. Statistical significance was set at $p<0.05$. All statistical analyses were performed with Stata/IC 13.1 (College Station, Texas, USA). This study is in accordance with the Strengthening the Reporting of Observational studies in Epidemiology (STROBE) Statement checklist (Supplementary 1) [21].

Qualitative part

FGDs were conducted among villagers and IDIs among healthcare workers. Three authors (K.I.C.O., P.K., S.Ke.) collected data until saturation was reached, indicating no new information emerging from the study participants even after additional focus group discussions or in-depth interviews were conducted.

FGD and IDI topic guides were created based on the quantitative questionnaire for the villagers. The FGD topic guide prompted the villagers to elaborate their experience with malaria, their choice of treatment, the reasons behind their decisions, health-seeking behaviors in general, and the cause of malaria. As for the healthcare workers, the IDI topic

For peer review only - http://bmjopen.bmj.com/site/about/guidelines.xhtml

For peer review only

guide asked about the health-seeking behaviors of the villagers, and the barriers in providing proper care.

All FGDs and IDIs were audio recorded and field notes were taken. Each villager and healthcare worker who finished the focus group discussion or in-depth interview was provided with a bag of sanitary items such as soap and washing detergents as an incentive. Audio data were transcribed verbatim in Lao language by native Lao research assistants. All the transcripts were double checked for accuracy. Thematic analysis was used to analyze the data and was performed by three authors (K.I.C.O., P.K., S.Ke.). To ensure trustworthiness in the analysis, the steps outlined by Nowell et al. [22] were followed. NVivo 12 Pro (QRS International Pty. Ltd, Doncaster, Australia) software was used to expedite the data analysis process. Guidelines in the Consolidated Criteria for Reporting Qualitative Research (COREQ) were followed (Supplementary 2) [23].

For peer review only

Data integration

Both the quantitative data and qualitative data were integrated to gain new insights through joint displays. A joint display is a visual means to combine both qualitative and quantitative data [24, 25].

Ethical considerations

Ethical approval was obtained from the Research Ethics Committee of the Graduate School of Medicine, The University of Tokyo (Serial number: 12038) and the National Ethics

For peer review only - http://bmjopen.bmj.com/site/about/guidelines.xhtml

Committee for Health Research, Ministry of Health, Lao PDR (No. 094/NECHR). Participation in this study was voluntary and the research assistants explained the purpose of the study prior to the data collection to the villagers and healthcare workers. A written informed consent was obtained from each participant. Personally identifiable information was not collected and the IDIs and FGDs were conducted in a secured place with low risk of being overheard by outsiders, such as the village meeting room and the meeting room of the health center.

Results

Quantitative part

Table 1 shows the sociodemographic characteristics of the villagers. In total, data were collected from 313 villagers; 202 from Village A and 111 from Village B. The number of female villagers in Village A was 128 (63.4%) while in Village B it was 44 (39.6%). In Village A, 198 (98.0%) villagers were Buddhists while in Village B, 81 (73.0%) were animists. The major ethnic group in Village A was Lao (197, 97.5%) while that of Village B was Katang (89, 80.2%).

[INSERT TABLE 1 HERE]

Table 2 shows the first place to seek treatment when a family member falls sick in both villages. Half of the villagers in Village A (101, 50.0%) would go to the health center first compared to 105 (94.6%) villagers in Village B.

For peer review only - http://bmjopen.bmj.com/site/about/guidelines.xhtml

For peer review only

[INSERT TABLE 2 HERE]

Table 3 shows the results of knowledge of malaria and treatment among the villagers. In Village A, 115 (56.9%) villagers answered that malaria is caused by mosquito bites compared to 85 (76.6%) villagers in Village B. Drinking dirty water was mentioned as a cause of malaria by 30 (14.9%) villagers in Village A and 27 (24.3%) villagers in Village B. Regarding protection against malaria, 150 (74.3%) villagers in Village A answered sleeping under an insecticide-treated bed net compared to 101 (91.0%) villagers in Village B. In Village A, 23 (11.4%) villagers thought that not drinking dirty water could protect oneself from malaria compared to 28 (25.2%) villagers in Village B.

[INSERT TABLE 3 HERE]

Table 4 shows the individual beliefs of the villagers in the context of malaria. In Village A, 109 (54.0%) villagers perceived that the consequences of malaria were serious compared to 60 (54.1%) villagers in Village B. Regarding perceived susceptibility, 112 (55.5%) villagers in Village A perceived that they were at high risk of malaria compared to 61 (55.0%) in Village B. As for perceived benefits, bed net use was thought to reduce malaria risk by 126 (62.4%) villagers in Village A compared to 71 (64.0%) villagers in Village B.

[INSERT TABLE 4 HERE]

Table 5 shows the responses of the villagers to clinical vignettes describing five conditions: hemolytic anemia, malaria, tuberculosis, acute respiratory infection, and diarrhea.

For peer review only

For peer review only - http://bmjopen.bmj.com/site/about/guidelines.xhtml

For malaria, 194 (96.0%) villagers in Village A responded that they would first seek treatment at public health facilities while 109 (98.2%) villagers in Village B responded that they would do the same. In Village B, 97-98% of the villagers answered that they would first seek treatment at the public health facilities for other conditions.

[INSERT TABLE 5 HERE]

Table 6 shows the factors associated with both perceived severity of malaria and perceived susceptibility of malaria. For perceived severity of malaria, those who have not experienced malaria before were more likely to perceive that the consequences of malaria were serious compared to those who have experienced malaria before (AOR=1.69, 95% CI: 1.03 to 2.75). Village, sex, ethnicity, malaria experience, and knowing about the cause of malaria were not significantly associated with perceived susceptibility.

[INSERT TABLE 6 HERE]

Qualitative part

In Village A, two male and two female FGDs were conducted while in Village B, three FGDs each for male and female villagers were conducted. The number of villagers per group was 3-4. Supplementaries 3 and 4 show the characteristics of the villagers who participated in the FGDs and healthcare workers who participated in the IDIs. The FGDs and IDIs lasted about an hour. Supplementary 5 shows the themes that emerged from the FGDs among the villagers.

For peer review only - http://bmjopen.bmj.com/site/about/guidelines.xhtml

For peer review only

Theme: Attributing the cause of malaria to something else other than being bitten by mosquitoes.

Some villagers believed that malaria was cause by something else other than mosquito bites.

"When I had malaria, they used 'pee pop' (ghost) to exorcize it away." (Village A, female, farmer)

"Drinking unclean water, you will get malaria." (Village B, male, farmer)

Theme: Describing an illness using local terms.

The villagers also described common illnesses in the area using local terms.

"'Kai luad niao' (sticky blood fever), when you are dehydrated, your blood will be sticky. When you drink water, your blood will dilute." (Village A, female, farmer)

"'Kai nyoong' (literally translated from Lao as 'mosquito fever', usually referred to malaria) in Katang language is called 'ae muay'" (Village B, female, farmer)

Theme: Expressing hope to improve the health center.

In the FGDs, the villagers expressed their hopes that the health center would be improved.

"I hope the staff will be more knowledgeable on the treatments." (Village B, male, farmer)

"I hope the village health center will be cleaner. There are mosquitoes here at the health center too, so you might even get malaria here!" (Village B, female, farmer)

Theme: Expressing difficulties in getting health services.

The villagers also mentioned about their difficulties in getting health services:

For peer review only - http://bmjopen.bmj.com/site/about/guidelines.xhtml

"'Kon luai bor dai kau kuk, kon tuk bor dai kau hong mor' (The rich do not go to jail, the poor do not go to the hospital)" (Village B, male, farmer)

"I hope the roads will also be improved. When it is raining, people die because they are not sent to the hospital in time." (Village B, male, farmer)

Theme: Mentioning about the National Health Insurance ("*Kor Por Sor*").

However, the villagers mentioned that the recently introduced National Health Insurance ("*Kor Por Sor*") was a facilitator in seeking treatment from the health center.

"I will go to the health center first, because I have to pay only 5000 kip. If I go to a private clinic, I will have to pay at least 50000 kip" (Village A, female, farmer)

Three IDIs were conducted in Village A (two for healthcare workers and one for village health volunteer) and four were conducted in Village B (three for healthcare workers and one for village health volunteer). Supplementary 6 shows the themes that emerged during the in-depth interviews with the healthcare workers.

Theme: Mentioning about the National Health Insurance ("*Kor Por Sor*").

When asked why the villagers come to the health centers, the healthcare workers also mentioned the National Health Insurance ("*Kor Por Sor*") as the facilitator.

"Now there is the 'Kor Por Sor' (National Health Insurance) system, where you have to pay only 5000 kip, they will come even when they have common cold." (Village A, female)

For peer review only

For peer review only - http://bmjopen.bmj.com/site/about/guidelines.xhtml

"Because of 'Kor Por Sor' (National Health Insurance), more people come to seek treatment at this health center." (Village B, male)

Theme: Expressing hope to improve the health center.

The healthcare workers also mentioned about their hopes to improve the health center.

"The knowledge and capability. I want to learn more. Another thing is the building. Too small. When people are sick they can't sleep here. If possible, I hope for more advanced equipment too." (Village B, male)

"At this health center the manpower is not enough, we also do not have enough knowledge. I hope people with knowledge will come here to work at this health center. I also hope this health center will be expanded. It is very crowded when there are many sick people and people who give birth." (Village B, female)

Data integration

Figures 1-3 shows the joint display combining both quantitative and qualitative data. Although in both villages, more than half of the villagers knew that malaria is caused by mosquito bites, some villagers still attributed the cause of malaria to drinking dirty water or water contaminated with mosquito larvae (Figure 1).

When probed about the first place to seek treatment when a family member is sick, 72.8% from Village A and 98.2% from Village B indicated that they would go to either the health center or district hospital. In the context of malaria, a vast majority, 88.6% from

For peer review only - http://bmjopen.bmj.com/site/about/guidelines.xhtml

Village A and 97.3% from Village B answered that they would go to the health center or the district hospital. In the qualitative part, both villagers and healthcare workers mentioned that after the introduction of the National Health Insurance ("*Kor Por Sor*"), the treatment cost became cheaper and this was the facilitator for the villagers to seek treatment at the health center or the district hospital (Figure 2).

Although most of the villagers seek treatment from the health center or the district hospital, and are confident to seek treatment when having malaria (98.0% from Village A and 96.4% from Village B), many problems and dissatisfactions exist. For example, the lack of medication and proper equipment and the attitude of the healthcare workers might discourage the villagers from seeking proper healthcare treatment (Figure 3).

Discussion

This study has three major findings. First, in the quantitative part, most of the villagers in both villages would seek treatment at the health center or the district hospital both in the context of malaria and other common diseases in the area. The level of knowledge of the villagers on the cause of malaria was low and the tendency to link malaria to causes other than mosquito bites was common. Second, in the qualitative part, both villagers and healthcare workers attributed increase in the number of villagers seeking treatment at the health center or the district hospital to the introduction of the National Health Insurance ("*Kor Por Sor*"). However, FGDs and IDIs revealed problems faced by the villagers in

For peer review only - http://bmjopen.bmj.com/site/about/guidelines.xhtml

seeking treatment and healthcare workers in providing care. Third, even though the introduction of the National Health Insurance ("*Kor Por Sor*") encouraged many villagers to seek treatment from the health center or the district hospital, both villagers and the healthcare workers were still facing hurdles in getting and providing optimal care.

In this study, villagers from both villages mainly sought treatment from the public healthcare providers: village health volunteer, health center, or the district hospital. In Lao PDR, health-seeking behaviors are influenced by several factors such as distance to a health center, socioeconomic factors, and family or friends' influence [26, 27]. Compared to another study in Nong District, Savannakhet Province [27], the number of villagers seeking treatment from traditional healers in this study was very low to none. The high usage of public healthcare providers in this study might be due to the ease in accessing these public healthcare providers as the health centers of both villages were situated in the villages [28]. Moreover, in another study in Lao PDR, people who initially visited a public healthcare provider were more likely to maintain a connection with the provider [29].

In the FGDs of the villagers and IDIs with the healthcare workers, one of the major reasons that increased the number of villagers seeking treatment at the health center or the district hospital was the introduction of the National Health Insurance ("*Kor Por Sor*"). In this scheme, villagers pay only 5000 kip to get basic treatment and medication at the health center and 10000 kip at the district hospital [15]. Patients only need to bring the family

For peer review only - http://bmjopen.bmj.com/site/about/guidelines.xhtml

registration book to prove their residence in the village. For those households without the family registration book, a special certificate issued by the village head is also acceptable [Personal communication with a Lao counterpart, based on an official Ministerial Order document]. In Lao PDR, although malaria diagnosis and treatment are free, patients had to pay for treatment of other illnesses and those from the low income bracket could not afford the treatment [30]. As economic status is a major barrier to getting proper treatment, the National Health Insurance has made it more affordable and accessible for the villagers to get treatment at the health center or the district hospital.

In this study, many villagers still attributed the cause of malaria to factors other than being bitten by mosquitoes, such as drinking unclean water. This result is consistent with another study among military personnel in Lao PDR [30]. Having knowledge on the cause of malaria does not necessarily lead to protective actions, but having knowledge can be a facilitator for self-implemented protective measures against malaria [31]. In the FGDs in both villages, many villagers described malaria and symptoms using local terms. This result emphasizes the need to tailor health messages and elimination strategies that are locally acceptable and understandable [11, 32].

In addition, villagers who have not experienced malaria before in this study were more likely to perceived malaria to be more severe than those who experienced before. Asymptomatic malaria carriers are very common in endemic areas in Lao PDR and these

For peer review only - http://bmjopen.bmj.com/site/about/guidelines.xhtml

carriers do not exhibit the usual malaria symptoms due to repeated infections [33, 34]. Thapangthong district was no exception and those who had experienced malaria before could be asymptomatic carriers. This result also highlights the necessity to tackle asymptomatic infections in Lao PDR in order to achieve the elimination goal.

Although the quantitative results showed high usage of health centers and the district hospital, both the FGDs with the villagers and IDIs with the healthcare workers revealed many barriers in treatment seeking and treatment delivering in the district. In this study, unavailability of medications and the lack of proper medical equipment were some of the major grievances of the villagers. Moreover, the current reporting system for malaria cases and antimalarial stocks at the village level to the district level in many malaria endemic areas in Lao PDR is still paper-based. This could cause a logistical delay in delivering the antimalarial stocks from Vientiane Capital to the endemic areas. In addition, some villagers also mentioned the unpleasant experience due to the attitude of the healthcare providers. Moreover, some of the healthcare providers acknowledged that they were not skilled enough in providing all treatments and hoped for better training. Lao PDR still faces many challenges in building a high-quality and equitable primary healthcare centers throughout the country [26]. The major challenges are difficult geographical terrains, inadequate training of healthcare staffs, lack of qualified health workers, low salaries, and poor morale among the staff [26, 35]. Therefore, although the current usage of the health center and the district

For peer review only - http://bmjopen.bmj.com/site/about/guidelines.xhtml

hospital is high, it is crucial to strengthen the capacity of the frontline healthcare facilities to monitor and eliminate malaria, improve the reporting mechanism by introducing online reporting system, and improve the overall health outcomes in the country.

This study has several strengths and limitations. This study provided evidence of the positive effect of the National Health Insurance ("*Kor Por Sor*"). Moreover, by using mixed methods where quantitative data and qualitative data are collected and interpreted together, this study provided a more comprehensive picture of the health-seeking behavior situation in Lao PDR, compared to using quantitative data or qualitative data alone.

However, the results of this study must be interpreted considering several limitations. First, in the FGDs and IDIs, social desirability might have affected the responses of both the villagers and healthcare workers. However, to obtain an honest response and to increase the credibility of the data, the researchers have established a good working relationship with the villagers and healthcare workers. Second, the results of this study are not representative of all malaria endemic districts of Lao PDR with different ethnic groups and different geographical terrains. However, transferability of the lessons learned from this study is possible by focusing on the key common issues affecting other malaria endemic areas such as lack of health personnel and the need to improve the healthcare facilities.

Conclusions

Although the treatment seeking in public healthcare facilities in this study was high,

For peer review only - http://bmjopen.bmj.com/site/about/guidelines.xhtml

the knowledge regarding malaria transmission was low. Moreover, both the villagers and healthcare providers expressed dissatisfaction with and the desire to improve the quality of the public healthcare facilities in this study. The introduction of the National Health Insurance ("*Kor Por Sor*") was regarded positively by both the villagers and the healthcare workers and this is a first step to achieving universal health coverage in Lao PDR. Therefore, a more integrated approach to improve healthcare seeking and healthcare delivery focusing on the villagers, healthcare providers, and the facilities should be accelerated.

Acknowledgements We would like to thank Dr. Chanthalangsouk Soukhavady, Dr. Syda Keothavone, Dr. Souphaphone Phimmasan, and Ms. Chansamone Khongkham for assisting in the data collection. Our gratitude also goes to the dedicated staff at the Center of Malaria Parasitology and Entomology, Savannakhet Provincial Health Office, and Thapangthong District Health Office for their support. We would also like to thank the villagers and healthcare workers who participated in this study.

Contributors KICO conceived and designed this study with the supervision from MJ. KICO, PK, SKe collected and analyzed the data. MI, PTB, SKa, and MJ provided critical comments and support during data collection and analysis. KICO wrote the first draft of the manuscript. All authors read and approved the final version of the manuscript.

For peer review only - http://bmjopen.bmj.com/site/about/guidelines.xhtml

Funding This study was financially supported by the JICA/AMED SATREPS project for the Development of innovative research techniques in the genetic epidemiology of malaria and other parasitic diseases in the Lao PDR for containing their expanding endemicity. The funder had no role in designing the study, collecting, analyzing and interpreting the data, preparing the manuscript, or decision to publish.

Competing interests None declared

Patient consent for publication Not required

Patient and public involvement No patients or members of the public were involved in the design, implementation, analyses, or reporting of the research.

Ethics approval This study was approved by the National Ethics Committee for Health Research (NECHR), National Institute of Public Health (NIOPH), Ministry of Health, Lao PDR (No. 049 NIOPH/NECHR) and the Research Ethics Committee of the University of Tokyo (No. 12038). Participation was voluntary and a written informed consent was obtained from all participants.

Data availability statement Data are available upon reasonable request to the corresponding author.

References

For peer review only

For peer review only - http://bmjopen.bmj.com/site/about/guidelines.xhtml

1. World Health Organization. Malaria. [https://www.who.int/en/news-room/fact-sheets/detail/malaria] Accessed 10 September 2019.
2. World Health Organization. World Malaria Report 2019. Geneva; 2019.
3. Feachem RGA, Chen I, Akbari O, Bertozzi-Villa A, Bhatt S, Binka F, et al. Malaria eradication within a generation: ambitious, achievable, and necessary. Lancet. 2019, 394:1056-1112.
4. Ghebreyesus TA. The malaria eradication challenge. Lancet. 2019, 394:990-991.
5. Hewitt S, Delacollette C, Chavez I. Malaria situation in the Greater Mekong Subregion. Southeast Asian J Trop Med Public Health. 2013, 44 Suppl 1:46-72; discussion 306-307.
6. Cui L, Yan G, Sattabongkot J, Cao Y, Chen B, Chen X, et al. Malaria in the Greater Mekong Subregion: heterogeneity and complexity. Acta Trop. 2012, 121:227-239.
7. Packard RM. The origins of antimalarial-drug resistance. N Engl J Med. 2014, 371:397-399.
8. Ministry of Health of the Lao People's Democratic Republic. National Strategy for Malaria Control and Elimination 2015-2020. Vientiane; 2015.
9. World Health Organization. Strategy for malaria elimination in the Greater Mekong Subregion (2015-2030). Geneva; 2015.
10. Messerli P, Heinimann A, Epprecht M, Phonesaly S, Thiraka C, Minot N. Socio-Economic Atlas of the Lao PDR an Analysis based on the 2005 Population and Housing Census. Swiss National Center of Competence in Research (NCCR) North-South and Geographica Bernensia; 2008.
11. Shirayama Y, Phompida S, Kuroiwa C. Modern medicine and indigenous health beliefs: malaria control alongside "Sadsana-phee" (animist belief system) in Lao PDR. Southeast Asian J Trop Med Public Health. 2006, 37:622-629.
12. Caillet C, Sichanh C, Syhakhang L, Delpierre C, Manithip C, Mayxay M, et al. Population awareness of risks related to medicinal product use in Vientiane Capital, Lao PDR: a cross-sectional study for public health improvement in low and middle income countries. BMC Public Health. 2015, 15:590.
13. Alvesson HM, Lindelow M, Khanthaphat B, Laflamme L. Coping with uncertainty during healthcare-seeking in Lao PDR. BMC Int Health Hum Rights. 2013, 13:28.
14. Tabernero P, Mayxay M, Culzoni MJ, Dwivedi P, Swamidoss I, Allan EL, et al. A Repeat Random Survey of the Prevalence of Falsified and Substandard Antimalarials in the Lao PDR: A Change for the Better. Am J Trop Med Hyg. 2015, 92:95-104.
15. World Health Organization. Lao Ministry of Health moves forward with National Health Insurance. [https://www.who.int/laos/news/detail/01-09-2019-lao-ministry-of-health-moves-forward-with-national-health-insurance] Accessed 5 December 2019.
16. Creswell JW. A concise introduction to mixed methods research. Los Angeles: Sage; 2015.

For peer review only

For peer review only - http://bmjopen.bmj.com/site/about/guidelines.xhtml

17. Ministry of Health and Lao Statistics Bureau. Lao social indicator survey 2011-12 (multiple indicator cluster survey/demographic and health survey). Vientiane: Ministry of Health and Lao Statistics Bureau; 2012.

18. RBM Partnership. Malaria Behavior Change Communication (BCC) Indicator Reference Guide. Geneva; 2014.

19. RBM Partnership. Malaria Social and Behavior Change Communication Indicator Reference Guide. 2nd ed. Geneva; 2017.

20. Mebratie AD, Van de Poel E, Yilma Z, Abebaw D, Alemu G, Bedi AS. Healthcare-seeking behaviour in rural Ethiopia: evidence from clinical vignettes. BMJ Open. 2014, 4:e004020.

21. von Elm E, Altman DG, Egger M, Pocock SJ, Gøtzsche PC, Vandenbroucke JP, et al. The Strengthening the Reporting of Observational Studies in Epidemiology (STROBE) statement: guidelines for reporting observational studies. PLoS Med. 2007, 4:e296.

22. Nowell LS, Norris JM, White DE, Moules NJ. Thematic Analysis: Striving to Meet the Trustworthiness Criteria. Int J Qual Methods. 2017, 16.

23. Tong A, Sainsbury P, Craig J. Consolidated criteria for reporting qualitative research (COREQ): a 32-item checklist for interviews and focus groups. Int J Qual Health Care. 2007, 19:349-357.

24. Fetters MD, Curry LA, Creswell JW. Achieving Integration in Mixed Methods Designs-Principles and Practices. Health Serv Res. 2013, 48:2134-2156.

25. Guetterman TC, Fetters MD, Creswell JW. Integrating Quantitative and Qualitative Results in Health Science Mixed Methods Research Through Joint Displays. Ann Fam Med. 2015, 13:554-561.

26. Bochaton A. Cross-border mobility and social networks: Laotians seeking medical treatment along the Thai border. Soc Sci Med. 2015, 124:364-373.

27. Adhikari B, Phommasone K, Pongvongsa T, Koummarasy P, Soundala X, Henriques G, et al. Treatment-seeking behaviour for febrile illnesses and its implications for malaria control and elimination in Savannakhet Province, Lao PDR (Laos): a mixed method study. BMC Health Serv Res. 2019, 19:252.

28. Suswardany DL, Sibbritt DW, Supardi S, Chang S, Adams J. A critical review of traditional medicine and traditional healer use for malaria and among people in malaria-endemic areas: contemporary research in low to middle-income Asia-Pacific countries. Malar J. 2015, 14:98.

29. Nonaka D, Vongseththa K, Kobayashi J, Bounyadeth S, Kano S, Phompida S, et al. Public and private sector treatment of malaria in Lao PDR. Acta Trop. 2009, 112:283-287.

30. Vilay P, Nonaka D, Senamonty P, Lao M, Iwagami M, Kobayashi J, et al. Malaria prevalence, knowledge, perception, preventive and treatment behavior among military in Champasak and Attapeu provinces, Lao PDR: a mixed methods study. Trop Med Health. 2019,

For peer review only - http://bmjopen.bmj.com/site/about/guidelines.xhtml

47.
31. Matsumoto-Takahashi EL, Tongol-Rivera P, Villacorte EA, Angluben RU, Yasuoka J, Kano S, et al. Determining the impact of community awareness-raising activities on the prevention of malaria transmission in Palawan, the Philippines. Parasitol Int. 2014, 63:519-526.
32. Nofal SD, Peto TJ, Adhikari B, Tripura R, Callery J, Bui TM, et al. How can interventions that target forest-goers be tailored to accelerate malaria elimination in the Greater Mekong Subregion? A systematic review of the qualitative literature. Malar J. 2019, 18:32.
33. Pongvongsa T, Nonaka D, Iwagami M, Nakatsu M, Phongmany P, Nishimoto F, et al. Household clustering of asymptomatic malaria infections in Xepon district, Savannakhet province, Lao PDR. Malar J. 2016, 15:508.
34. Iwagami M, Keomalaphet S, Khattignavong P, Soundala P, Lorphachan L, Matsumoto-Takahashi E, et al. The detection of cryptic Plasmodium infection among villagers in Attapeu province, Lao PDR. PLoS Negl Trop Dis. 2017, 11:e0006148.
35. Kounnavong S, Gopinath D, Hongvanthong B, Khamkong C, Sichanthongthip O. Malaria elimination in Lao PDR: the challenges associated with population mobility. Infect Dis Poverty. 2017, 6:81.

For peer review only

For peer review only - http://bmjopen.bmj.com/site/about/guidelines.xhtml

Table 1 Sociodemographic characteristics of the villagers

	Village A	Village B
Villagers interviewed	202	111
Sex, n (%)		
Male	74 (36.6)	67 (60.4)
Female	128 (63.4)	44 (39.6)
Age		
Minimum	18	18
Maximum	85	73
Median	40	39
Mean	40.9	38.6
Religion, n (%)		
Buddhism	198 (98.0)	30 (27.0)
Animism	4 (2.0)	81 (73.0)
Ethnicity, n (%)		
Lao	197 (97.5)	21 (18.9)
Katang	2 (1.0)	89 (80.2)
Do not know	3 (1.5)	1 (0.9)
Marital status, n (%)		
Married	183 (90.6)	97 (87.4)
Single	5 (2.5)	6 (5.4)
Divorced	2 (1.0)	1 (0.9)
Widowed	12 (5.9)	6 (5.4)
Others	0	1 (0.9)
Occupation, n (%)		
Farmer	170 (84.2)	105 (94.6)
Soldier	1 (0.5)	0
Housewife	18 (8.9)	2 (1.8)
Businessman/women	1 (0.5)	1 (0.9)
Government servant	7 (3.5)	2 (1.8)

For peer review only

For peer review only - http://bmjopen.bmj.com/site/about/guidelines.xhtml

Table 1 Sociodemographic characteristics of the villagers (continued)

	Village A	Village B
Others	5 (2.5)	1 (0.9)
Education, n (%)		
No education	71 (35.2)	42 (37.8)
Preschool	1 (0.5)	1 (0.9)
Primary school	84 (41.6)	46 (41.4)
Lower secondary school	28 (13.9)	14 (12.6)
Upper secondary school	8 (4.0)	5 (4.5)
Post secondary non-tertiary	8 (4.0)	2 (1.8)
Tertiary	2 (1.0)	1 (0.9)
Average monthly household income (kip) (US$1=8000 kip [Jan 2019])		
Minimum	0	0
Maximum	10000000	4000000
Median	200000	100000
Mean	496881	290000
Number of household members (Median)	5	6
Have insecticide-treated nets at home		
Yes	169 (83.7)	107 (96.4)
No	31 (15.4)	4 (3.6)
Don't know	2 (1.0)	0

**Percentage may not add up to 100 due to rounding*

For peer review only - http://bmjopen.bmj.com/site/about/guidelines.xhtml

Table 2 First place to seek treatment when a family member falls sick

Where to seek treatment, n (%)	Village A (n=202)	Village B (n=111)
Village health volunteer	25 (12.4)	0
Health center	101 (50.0)	105 (94.6)
District hospital	46 (22.8)	4 (3.6)
Provincial hospital	6 (3.0)	1 (0.9)
Private pharmacy	10 (5.0)	0
Traditional healer	1 (0.5)	0
Others	13 (6.4)	1 (0.9)

For peer review only

For peer review only - http://bmjopen.bmj.com/site/about/guidelines.xhtml

Table 3 Knowledge of malaria and treatment

	Village A (n=202)	Village B (n=111)
What causes malaria? n (%)		
Mosquito bites	115 (56.9)	85 (76.6)
Eating dirty food	17 (8.4)	9 (8.1)
Eating uncooked food	6 (3.0)	7 (6.3)
Drinking dirty water	30 (14.9)	27 (24.3)
Witchcraft	0	0
Going to the forest	73 (36.1)	49 (44.1)
Changing weather	8 (4.0)	3 (2.7)
Getting soaked with rain	3 (1.5)	1 (0.9)
How to protect yourself against malaria? n (%)		
Sleep under an insecticide-treated mosquito net	150 (74.3)	101 (91.0)
Use mosquito repellent	9 (4.5)	4 (3.6)
Avoid mosquito bites	9 (4.5)	5 (4.5)
Avoid going to the forest	10 (5.0)	4 (3.6)
Take preventive medicine	2 (1.0)	0
Spray house with insecticide	8 (4.0)	1 (0.9)
Use mosquito coils	10 (5.0)	4 (3.6)
Fill in puddles around the house	18 (8.9)	3 (2.7)
Keep surrounding of the house clean	54 (26.7)	42 (37.8)
Burn leaves	4 (2.0)	10 (9.0)
Do not drink dirty water	23 (11.4)	28 (25.2)
Do not eat dirty food	8 (4.0)	12 (10.8)
Put mosquito screens on the window	0	1 (0.9)
Do not get soaked with rain water	0	0
What are the symptoms that indicate a person has malaria? n (%)		
Fever	115 (56.9)	70 (63.1)
Feeling cold	89 (44.1)	73 (65.8)
Headache	116 (57.4)	85 (76.6)
Nausea and vomiting	30 (14.9)	17 (15.3)
Diarrhea	2 (1.0)	2 (1.8)
Dizziness	16 (7.9)	13 (11.7)

For peer review only - http://bmjopen.bmj.com/site/about/guidelines.xhtml

Loss of appetite	12 (5.9)	4 (3.6)
Bodyache/Joint pain	58 (28.7)	23 (20.7)
Pale eyes	5 (2.5)	0
Feeling weak	14 (6.9)	9 (8.1)
What is the most effective medication to treat malaria? n (%)		
Chloroquine	0	1 (0.9)
Quinine	2 (1.0)	3 (2.7)
Artemisinin combination therapy (Coartem TM)	3 (1.5)	12 (10.8)
Aspirin, panadol, paracetamol	12 (5.9)	8 (7.2)
Do not know	185 (91.6)	85 (76.6)
What are the main danger signs of malaria? n (%)		
Seizure	29 (14.4)	16 (14.4)
Fainting	7 (3.5)	5 (4.5)
Any fever	8 (4.0)	1 (0.9)
High fever	26 (12.9)	20 (18.0)
Stiff neck	2 (1.0)	3 (2.7)
Feeling weak	8 (4.0)	3 (2.7)
Not active	2 (1.0)	2 (1.8)
Chills/shivering	30 (14.9)	24 (21.6)
Not able to eat	4 (2.0)	1 (0.9)
Vomiting	9 (4.5)	18 (16.2)
Crying all the time	4 (2.0)	1 (0.9)
Restless	19 (9.4)	14 (12.6)
Diarrhea	0	2 (1.8)

For peer review only - http://bmjopen.bmj.com/site/about/guidelines.xhtml

Table 4 Individual beliefs

		Village A (n=202)	Village B (n=111)	p-value
Villagers who perceived the consequences of malaria were serious (Perceived severity) n (%)		109 (54.0)	60 (54.1)	0.987
Villagers who perceived they were at high risk of malaria (Perceived susceptibility) n (%)		112 (55.5)	61(55.0)	0.933
Villagers who believed that the recommended practice or product will reduce their risk (Perceived benefits) n (%)				
	Bed net use	126 (62.4)	71 (64.0)	0.781
	Malaria diagnostics	120 (59.4)	64 (57.7)	0.764
	Malaria treatments with ACT	159 (78.7)	88 (79.3)	0.906
Villagers who are confident in their abilities to perform a specific malaria-related behavior (Self-efficacy) n (%)				
	Protection of self and family	158 (78.2)	76 (68.5)	0.057
	Bed net use	198 (98.0)	110 (99.1)	0.466
	Malaria detection	153 (75.7)	81 (73.0)	0.589
	Seek diagnosis	185 (91.6)	96 (86.5)	0.154
	Seek treatment	198 (98.0)	107 (96.4)	0.384

Chi-square test; ACT=Artemisinin combination therapy

For peer review only - http://bmjopen.bmj.com/site/about/guidelines.xhtml

For peer review only

Table 5 Clinical vignettes: First place to seek treatment

	Hemolytic anemia (n (%))		Malaria (n (%))		Tuberculosis (n (%))		Acute respiratory infection (n (%))		Diarrhea (n (%))	
	Village A (n=202)	Village B (n=111)	Village A (n=202)	Village B (n=111)	Village A (n=202)	Village B (n=111)	Village A (n=202)	Village B (n=111)	Village A (n=202)	Village B (n=111)
Public health facilities	190 (94.1)	109 (98.2)	194 (96.0)	109 (98.2)	189 (93.6)	108 (97.3)	181 (89.6)	109 (98.2)	188 (93.1)	108 (97.3)
Others	9 (4.5)	1 (0.9)	7 (3.5)	1 (0.9)	11 (5.5)	2 (1.8)	20 (9.9)	1 (0.9)	12 (5.9)	2 (1.8)
Take no action	3 (1.5)	1 (0.9)	1 (0.5)	1 (0.9)	2 (1.0)	1 (0.9)	1 (0.5)	1 (0.9)	2 (1.0)	1 (0.9)

For peer review only

Table 6 Factors associated with perceived severity and perceived susceptibility

Perceived severity of malaria

Characteristics		Adjusted Odds Ratio (95% CI)	p-value
Village			
	Village A	1.00	0.811
	Village B	1.11 (0.48-2.58)	
Sex			
	Male	1.00	0.090
	Female	0.65 (0.40-1.07)	
Ethnicity			
	Lao	1.00	0.615
	Others	0.80 (0.34-1.89)	
Experienced malaria before			
	Yes	1.00	**0.036**
	No	1.69 (1.03-2.75)	
Knowing that malaria is caused by mosquito bites			
	Yes	1.00	0.217
	No	0.74 (0.46-1.20)	

Perceived susceptibility of malaria

Characteristics		Adjusted Odds Ratio (95% CI)	p-value
Village			
	Village A	1.00	0.805
	Village B	0.90 (0.39-2.07)	
Sex			
	Male	1.00	0.910
	Female	1.03 (0.63-1.67)	
Ethnicity			
	Lao	1.00	0.754
	Others	1.14 (0.49-2.67)	
Experienced malaria before			
	Yes	1.00	0.344
	No	1.26 (0.78-2.04)	
Knowing that malaria is caused by mosquito bites			
	Yes	1.00	0.611
	No	0.88 (0.55-1.43)	

1

For peer review only - http://bmjopen.bmj.com/site/about/guidelines.xhtml

For peer review only

Supplementary 1 STROBE Statement—Checklist of items that should be included in reports of ***cross-sectional studies***

	Item No	Recommendation	Page No
**Title and abstract**	1	(*a*) Indicate the study's design with a commonly used term in the title or the abstract	1
		(*b*) Provide in the abstract an informative and balanced summary of what was done and what was found	2
**Introduction**			
Background/rationale	2	Explain the scientific background and rationale for the investigation being reported	5-7
Objectives	3	State specific objectives, including any prespecified hypotheses	5-7
**Methods**			
Study design	4	Present key elements of study design early in the paper	7
Setting	5	Describe the setting, locations, and relevant dates, including periods of recruitment, exposure, follow-up, and data collection	7-8
Participants	6	(*a*) Give the eligibility criteria, and the sources and methods of selection of participants	8
Variables	7	Clearly define all outcomes, exposures, predictors, potential confounders, and effect modifiers. Give diagnostic criteria, if applicable	9-10
Data sources/ measurement	8*	For each variable of interest, give sources of data and details of methods of assessment (measurement). Describe comparability of assessment methods if there is more than one group	9-10
Bias	9	Describe any efforts to address potential sources of bias	22
Study size	10	Explain how the study size was arrived at	8
Quantitative variables	11	Explain how quantitative variables were handled in the analyses. If applicable, describe which groupings were chosen and why	9-10
Statistical methods	12	(*a*) Describe all statistical methods, including those used to control for confounding	9-10
		(*b*) Describe any methods used to examine subgroups and interactions	NA
		(*c*) Explain how missing data were addressed	NA
		(*d*) If applicable, describe analytical methods taking account of sampling strategy	NA
		(*e*) Describe any sensitivity analyses	NA
**Results**			
Participants	13*	(a) Report numbers of individuals at each stage of study—eg numbers potentially eligible, examined for eligibility, confirmed eligible, included in the study, completing follow-up, and analysed	12-14
		(b) Give reasons for non-participation at each stage	NA
		(c) Consider use of a flow diagram	NA
Descriptive data	14*	(a) Give characteristics of study participants (eg demographic, clinical, social) and information on exposures and potential confounders	12-14
		(b) Indicate number of participants with missing data for each variable of interest	NA
Outcome data	15*	Report numbers of outcome events or summary measures	12-14

For peer review only - http://bmjopen.bmj.com/site/about/guidelines.xhtml

Main results	16	(*a*) Give unadjusted estimates and, if applicable, confounder-adjusted estimates and their precision (eg, 95% confidence interval). Make clear which confounders were adjusted for and why they were included	14
		(*b*) Report category boundaries when continuous variables were categorized	NA
		(*c*) If relevant, consider translating estimates of relative risk into absolute risk for a meaningful time period	NA
Other analyses	17	Report other analyses done—eg analyses of subgroups and interactions, and sensitivity analyses	NA
**Discussion**			
Key results	18	Summarise key results with reference to study objectives	18-19
Limitations	19	Discuss limitations of the study, taking into account sources of potential bias or imprecision. Discuss both direction and magnitude of any potential bias	22
Interpretation	20	Give a cautious overall interpretation of results considering objectives, limitations, multiplicity of analyses, results from similar studies, and other relevant evidence	18-22
Generalisability	21	Discuss the generalisability (external validity) of the study results	22
**Other information**			
Funding	22	Give the source of funding and the role of the funders for the present study and, if applicable, for the original study on which the present article is based	24

*Give information separately for exposed and unexposed groups.

Note: An Explanation and Elaboration article discusses each checklist item and gives methodological background and published examples of transparent reporting. The STROBE checklist is best used in conjunction with this article (freely available on the Web sites of PLoS Medicine at http://www.plosmedicine.org/, Annals of Internal Medicine at http://www.annals.org/, and Epidemiology at http://www.epidem.com/). Information on the STROBE Initiative is available at www.strobe-statement.org.

For peer review only

Supplementary 2 Consolidated criteria for reporting qualitative studies (COREQ): 32-item checklist

Developed from:
Tong A, Sainsbury P, Craig J. Consolidated criteria for reporting qualitative research (COREQ): a 32-item checklist for interviews and focus groups. *International Journal for Quality in Health Care*. 2007. Volume 19, Number 6: pp. 349 – 357

No. Item	Guide questions/description	Reported on Page #
**Domain 1: Research team and reflexivity**		
Personal Characteristics		
1. Inter viewer/facilitator	Which author/s conducted the interview or focus group?	10
2. Credentials	What were the researcher's credentials? E.g. PhD, MD	9
3. Occupation	What was their occupation at the time of the study?	9
4. Gender	Was the researcher male or female?	NA
5. Experience and training	What experience or training did the researcher have?	9
Relationship with participants		
6. Relationship established	Was a relationship established prior to study commencement?	22
7. Participant knowledge of the interviewer	What did the participants know about the researcher? e.g. personal goals, reasons for doing the research	8
8. Interviewer characteristics	What characteristics were reported about the inter viewer/facilitator? e.g. Bias, assumptions, reasons and interests in the research topic	NA
**Domain 2: study design**		
Theoretical framework		
9. Methodological orientation and Theory	What methodological orientation was stated to underpin the study? e.g. grounded theory, discourse analysis, ethnography, phenomenology, content analysis	10-11
Participant selection		
10. Sampling	How were participants selected? e.g. purposive, convenience, consecutive, snowball	8
11. Method of approach	How were participants approached? e.g. face-to-face, telephone, mail, email	8
12. Sample size	How many participants were in the study?	14
13. Non-participation	How many people refused to participate or dropped out? Reasons?	NA
Setting		
14. Setting of data collection	Where was the data collected? e.g. home, clinic, workplace	12

For peer review only - http://bmjopen.bmj.com/site/about/guidelines.xhtml

15. Presence of non-participants	Was anyone else present besides the participants and researchers?	NA
16. Description of sample	What are the important characteristics of the sample? e.g. demographic data, date	14
Data collection		
17. Interview guide	Were questions, prompts, guides provided by the authors? Was it pilot tested?	10
18. Repeat interviews	Were repeat inter views carried out? If yes, how many?	NA
19. Audio/visual recording	Did the research use audio or visual recording to collect the data?	11
20. Field notes	Were field notes made during and/or after the inter view or focus group?	11
21. Duration	What was the duration of the inter views or focus group?	14
22. Data saturation	Was data saturation discussed?	10
23. Transcripts returned	Were transcripts returned to participants for comment and/or correction?	NA
**Domain 3: analysis and findings**		
Data analysis		
24. Number of data coders	How many data coders coded the data?	11
25. Description of the coding tree	Did authors provide a description of the coding tree?	NA
26. Derivation of themes	Were themes identified in advance or derived from the data?	11
27. Software	What software, if applicable, was used to manage the data?	11
28. Participant checking	Did participants provide feedback on the findings?	NA
Reporting		
29. Quotations presented	Were participant quotations presented to illustrate the themes/findings? Was each quotation identified? e.g. participant number	15-17
30. Data and findings consistent	Was there consistency between the data presented and the findings?	15-17
31. Clarity of major themes	Were major themes clearly presented in the findings?	15-17
32. Clarity of minor themes	Is there a description of diverse cases or discussion of minor themes?	15-17

For peer review only

Supplementary 3 Characteristics of the villagers in the focus group discussions

No.	Village	Content	ID	Unique ID	Participants
1	A	Male FGD	5.59	BM1.1	Retiree
			2.67	BM1.2	Teacher
			2.112	BM1.3	Government servant
			4.113	BM1.4	Soldier
2	A	Male FGD	6.69	BM2.1	Farmer
			5.126	BM2.2	Farmer
			5.129	BM2.3	Farmer
3	A	Female FGD	5.6	BF1.1	Businesswoman
			6.50	BF1.2	Farmer
			2.102	BF1.3	Farmer
4	A	Female FGD	1.122	BF2.1	Farmer
			4.139	BF2.2	Farmer
			3.140	BF2.3	Government servant
5	B	Male FGD	3.61	NM1.1	Government servant
			3.76	NM1.2	Farmer
			4.77	NM1.3	Farmer
6	B	Male FGD	4.87	NM2.1	Farmer
			3.89	NM2.2	Farmer
			1.91	NM2.3	Farmer
7	B	Male FGD	2.93	NM3.1	Farmer
			1.95	NM3.2	Farmer
			1.102	NM3.3	Farmer
8	B	Female FGD	5.41	NF1.1	Farmer
			1.54	NF1.2	Farmer
			2.55	NF1.3	Farmer
9	B	Female FGD	1.98	NF2.1	Farmer
			2.101	NF2.2	Farmer
			3.106	NF2.3	Farmer
10	B	Female FGD	3.99	NF3.1	Farmer
			4.100	NF3.2	Farmer
			2.104	NF3.3	Farmer

For peer review only

For peer review only - http://bmjopen.bmj.com/site/about/guidelines.xhtml

Supplementary 4 Characteristics of the healthcare workers in the in-depth interviews

No.	Village	Content	Unique ID	Age	Sex
1	A	Healthcare worker IDI	BH1		Female
2	A	Healthcare worker IDI	BH2		Female
3	A	Village Health Volunteer IDI	BV1		Male
4	B	Healthcare worker IDI	NH1		Male
5	B	Healthcare worker IDI	NH2		Female
6	B	Healthcare worker IDI	NH3		Female
7	B	Village Health Volunteer IDI	NV1		Male

For peer review only

For peer review only - http://bmjopen.bmj.com/site/about/guidelines.xhtml

Supplementary 5 Themes from the focus group discussions of the villagers

Themes	Quotes, Unique ID
Attributing the cause of malaria to something else other than being bitten by mosquitoes	1. Because your house is not clean. If you drink water with mosquito eggs, you will get fever. *BF 2.3 Government servant*<br>2. When I had malaria, they used '*pee pop*' (ghost) to exorcize it away. *BF 2.1 Farmer*<br>3. Drinking unclean water. *BM1.4 Soldier*<br>4. Drinking unclean water, you will get malaria. *NM 2.3 Farmer*<br>5. Scared of malaria because scared of '*pop hed*' (caused by ghost). *NF1.2 Farmer*
Attributing the cause of malaria to being bitten by mosquitoes	1. Mosquito bite, not sleeping under the bed net, not taking care of oneself. Not protecting oneself. *BF1.1 Businesswoman*<br>2. Caused by mosquitoes. You must be bitten by mosquitoes first. *BF 2.1 Farmer*<br>3. Because of mosquito bites. When you do not sleep under the bed net, the mosquitoes will bite you. *NM1.1 Government servant*<br>4. Do not sleep under the bed net, being bitten by mosquitoes. *NM3.2 Farmer*
Describing an illness using local terms	1. '*Kai luad niao*' (sticky blood fever), when you are dehydrated, your blood will be sticky. When you drink water, your blood will dilute. *BF1.3 Farmer*<br>2. Among men, '*kai luad niao*' (sticky blood fever) is also very common. There were two cases this year, and they died. *BM1.4 Soldier*<br>3. '*Kai nyoong*' (literally translated from Lao as 'mosquito fever', usually referred to malaria) in Katang language is called '*ae muay*'. *NF1.1 Farmer*
Expressing hope to improve the health	1. I just hope the health center will be well-equipped with medicines and such, and I hope things will get better. I am not criticizing them,

For peer review only - http://bmjopen.bmj.com/site/about/guidelines.xhtml

center		I understand that there is not enough budget. *BF1.3 Farmer*
	2.	I hope the health center will be better equipped, with Echo machine, with qualified medical doctors, surgical services. *NM1.2 Farmer*
	3.	I hope the staff will be more knowledgeable on the treatments. *NM3.2 Farmer*
	4.	Improve the health center, I hope they have X-ray machines, proper equipment. *NF1.1 Farmer*
	5.	I hope there will be more medical personnel from other villages stationed here and live here so it will be convenient for us to call on them. *NF1.1 Farmer*
	6.	I hope the health center will be cleaner. There are mosquitoes here at the health center too, so you might even get malaria here! *NF2.2 Farmer*
	7.	I hope the staff at the health center will further their studies and improve their knowledge to help the villagers become healthy. *NM3.1, Farmer*
Expressing hope to improve the district hospital	1.	I hope there will also be a new building at the district hospital. Sometimes there are not enough beds for the patients and the family members have no place to stay. *NM1.1 Government servant; NM1.2 Farmer; NM1.3 Farmer*
Recalling personal experience	1.	We do not have oxygen here in the district hospital. I gave birth to a premature baby once and it died because we did not have oxygen. We were going to the Savannakhet provincial hospital and the baby died on the way in Pakxong. No incubator too. *BF1.1 Businesswoman*
	2.	When I got fever and the health staff gave me the wrong medicine. They do not know. *NM3.3 Farmer*
Expressing opinion on the health center	1.	Overall everything is good but only one thing is that medicine is always not enough. *BM1.2 Teacher*
	2.	Sometimes, when people are shabbily dressed, the staff do not want

For peer review only

	to treat them. *BM1.4 Soldier*<br><br>3. If you pay less you get only 'paracetamol'. You won't get well. Last time I had to pay but I felt I recovered fast. Now I pay only 5000 kip I don't get well soon. *NF2.1 Farmer; NF2.2 Farmer; NF2.3 Farmer*
Feeling towards malaria	1. Mosquitoes are scarier than tigers. *NM1.2 Farmer*<br><br>2. Scary! Mosquitoes are scarier than tigers. *NM3.1 Farmer*
Expressing opinion towards malaria elimination	1. Yes, malaria can be eliminated. Destroy the mosquitoes. Spray them. They will be gone. *BF1.3 Farmer*<br><br>2. Elimination is difficult because when people go to the forest, they drink water from the stream. *BF 2.3 Government servant*<br><br>3. It's difficult to eliminate malaria. The only way is to treat. *BM1.4 Soldier*<br><br>4. Cannot be eliminated because our people depend on the forest to make a living, they will just sleep when it gets dark when they are in the forest. *BM2.3 Farmer*<br><br>5. Yes, take care of hygiene, protect oneself when going to the forest. *BM1.4 Soldier*
Expressing difficulties in getting health services	1. '*Kon luai bor dai kau kuk, kon tuk bor dai kau hong mor*' (The rich do not go to jail, the poor do not go to the hospital). *NM1.3, Farmer*<br><br>2. I hope the roads will also be improved. When it is raining, people die because they are not sent to the hospital in time. *NM1.2 Farmer*
Mentioning about the National Health Insurance ("*Kor Por Sor*")	1. I will go to the health center first, because I have to pay only 5000 kip. If I go to a private clinic, I will have to pay at least 50000 kip. *BF 2.1 Farmer*

For peer review only - http://bmjopen.bmj.com/site/about/guidelines.xhtml

Supplementary 6 Themes from the in-depth interviews of the healthcare workers

Themes	Quotes, Unique ID
Mentioning about the National Health Insurance ("*Kor Por Sor*")	1. Now there is the *'Kor Por Sor'* (National Health Insurance) system, where you have to pay only 5000 kip, they will come even when they have common cold. *BH1, Female*<br>2. Because of *'Kor Por Sor'* (National Health Insurance), more people come to seek treatment at this health center. *NH1, Male*<br>3. Because of *'Kor Por Sor'* (National Health Insurance), we have more patients who come here for treatment. *NH2, Female*
Expressing opinion towards malaria elimination	1. Yes, you can eliminate malaria. You should sleep under the long-lasting insecticide-treated bed nets. In addition, we should also train the village health volunteers to use the rapid diagnostic test (RDT) so we can eliminate malaria. *BH1, Female*<br>2. It's difficult to eliminate because people here make a living by going to the mountains, to the forests. We can't stop them from going. They need to make a living. *NH2, Female*<br>3. You can't eliminate 100%. People here work in the field, go to the forest, go to the mountains. *NH3, Female*
Describing an illness using local terms	1. I heard the villagers mentioned '*luad niao*', '*luad niao*', is there such thing? *BH2, Female*
Describing the villagers' health-seeking behavior	1. Yes, when they fall sick, they will go to the '*mor pee*' (witch doctor) first. But only for some families. Usually the old. *BV1, Male*<br>2. The villagers come to the health center directly as we do not encourage them to go the village health volunteer first. I am afraid that the village health volunteer do not know the right way to treat the patients. *BH2, Female*
Feeling towards the job	1. I am proud of my work. I am a nurse but I also function as a 'doctor' here. I have to work more than what I studied. It is very challenging sometimes but the longer I work, the more experience I have. *NH2, Female*<br>2. I am satisfied with my work. Because this work can save lives. *NV1, Male*
Expressing hope to improve the health center	1. The knowledge and capability. I want to learn more. Another thing is the building. Too small. When people are sick they can't sleep here. If possible, I hope for more advanced equipment too. *NH1, Male*

For peer review only - http://bmjopen.bmj.com/site/about/guidelines.xhtml

	2. I hope the health center will be expanded. I also want to have machines that can check blood or Echo machine. I also hope that a medical doctor will be stationed here. *NH2, Female*<br>3. At this health center the manpower is not enough, we also do not have enough knowledge. I hope people with knowledge will come here to work at this health center. I also hope this health center will be expanded. It is very crowded when there are many sick people and people who give birth. *NH3, Female*
Expressing hope to improve the living condition of the people	1. I hope there will be toilets in every house. *NV1, Male*

For peer review only

For peer review only - http://bmjopen.bmj.com/site/about/guidelines.xhtml

BMJ Open

Health-seeking behaviors in a malaria endemic district in Lao People's Democratic Republic: a mixed methods study

Journal:	*BMJ Open*
Manuscript ID	bmjopen-2021-055350.R1
Article Type:	Original research
Date Submitted by the Author:	15-Oct-2021

Complete List of Authors:	Ong, Ken; The University of Tokyo, Department of Community and Global Health<br>Khattignavong, Phonepadith; Institut Pasteur du Laos<br>Keomalaphet, Sengdeuane; Institut Pasteur du Laos<br>Iwagami, Moritoshi; Institut Pasteur du Laos; National Center for Global Health and Medicine Research Institute, Department of Tropical Medicine and Malaria<br>Brey, Paul; Institut Pasteur du Laos<br>Kano, Shigeyuki; National Center for Global Health and Medicine Research Institute, Department of Tropical Medicine and Malaria<br>Jimba, Masamine; The University of Tokyo, Community and Global Health

**Primary Subject Heading**:	Global health
Secondary Subject Heading:	Infectious diseases
Keywords:	Public health < INFECTIOUS DISEASES, PUBLIC HEALTH, QUALITATIVE RESEARCH

SCHOLARONE™
Manuscripts

I, the Submitting Author has the right to grant and does grant on behalf of all authors of the Work (as defined in the below author licence), an exclusive licence and/or a non-exclusive licence for contributions from authors who are: i) UK Crown employees; ii) where BMJ has agreed a CC-BY licence shall apply, and/or iii) in accordance with the terms applicable for US Federal Government officers or employees acting as part of their official duties; on a worldwide, perpetual, irrevocable, royalty-free basis to BMJ Publishing Group Ltd ("BMJ") its licensees and where the relevant Journal is co-owned by BMJ to the co-owners of the Journal, to publish the Work in this journal and any other BMJ products and to exploit all rights, as set out in our licence.

The Submitting Author accepts and understands that any supply made under these terms is made by BMJ to the Submitting Author unless you are acting as an employee on behalf of your employer or a postgraduate student of an affiliated institution which is paying any applicable article publishing charge ("APC") for Open Access articles. Where the Submitting Author wishes to make the Work available on an Open Access basis (and intends to pay the relevant APC), the terms of reuse of such Open Access shall be governed by a Creative Commons licence – details of these licences and which Creative Commons licence will apply to this Work are set out in our licence referred to above.

Other than as permitted in any relevant BMJ Author's Self Archiving Policies, I confirm this Work has not been accepted for publication elsewhere, is not being considered for publication elsewhere and does not duplicate material already published. I confirm all authors consent to publication of this Work and authorise the granting of this licence.

For peer review only

Title: Health-seeking behaviors in a malaria endemic district in Lao People's Democratic Republic: a mixed methods study

Ken Ing Cherng Ong[1,2*], Phonepadith Khattignavong[2,3], Sengdeuane Keomalaphet[2,3], Moritoshi Iwagami[2,3,4], Paul T. Brey[2,3], Shigeyuki Kano[2,3,4], Masamine Jimba[1,2]

[1]Department of Community and Global Health, Graduate School of Medicine, The University of Tokyo, Tokyo, Japan

[2]SATREPS project (JICA/AMED) for Parasitic Diseases, Vientiane, Lao PDR

[3]Institut Pasteur du Laos, Ministry of Health, Samsenthai Road, Ban Kao-Gnot, Sisattanak District, P.O. Box 3560, Vientiane, Lao PDR

[4]Department of Tropical Medicine and Malaria, Research Institute, National Centre for Global Health and Medicine, 1-21-1 Toyama, Shinjuku, Tokyo 162-8655, Japan

***Corresponding author: Ken Ing Cherng Ong (kenicong@m.u-tokyo.ac.jp)**

Word counts: 4649 words

For peer review only - http://bmjopen.bmj.com/site/about/guidelines.xhtml

Abstract

Objectives: This mixed methods study was conducted to explore the barriers and facilitators for health-seeking behaviors in a malaria endemic district in Lao PDR.

Design: A convergent mixed methods design.

Setting: Two malaria endemic villages in Thapangthong district, Savannakhet Province, Lao PDR.

Participants: Villagers and healthcare workers in the two villages in Thapangthong district.

Methods: In the quantitative part, a pre-tested questionnaire was used to identify the health-seeking behaviors of the villagers. In the qualitative part, focus group discussions were employed to explore health-seeking behaviors of the villagers and in-depth interviews were used to explore the perceptions of the healthcare workers. Descriptive statistics were computed and multiple logistic regressions were used to identify the factors associated with perceived severity and perceived susceptibility. Thematic analysis was used to analyze the qualitative data. Quantitative and qualitative results were integrated in joint displays.

Results: In the quantitative part, data were collected from 313 villagers from both villages. For malaria, 96.0% and 98.2% of villagers from Villages A and B respectively would first seek treatment at public health facilities. Villagers who have not experienced malaria before were more likely to perceive that the consequences of malaria were serious compared to those who have experienced malaria before (AOR=1.69, 95% CI: 1.03 to 2.75). However,

For peer review only - http://bmjopen.bmj.com/site/about/guidelines.xhtml

qualitative data showed that villagers faced problems such as lack of medicines and medical equipment. Healthcare workers also mentioned the lack of manpower and equipment in the in-depth interviews. Nevertheless, villagers still preferred to seek treatment at the health center as the National Health Insurance was introduced.

Conclusions: Public health facility usage was high but barriers existed. Effective policy and enabling environment such as the introduction of the National Health Insurance could help accelerate the progress towards the malaria elimination goal. Moreover, the benefits could go beyond the context of malaria.

For peer review only

For peer review only - http://bmjopen.bmj.com/site/about/guidelines.xhtml

Strengths and limitations of this study

- This study used both quantitative and qualitative methods to explore the health-seeking behaviors in a malaria endemic district in Lao PDR.
- Perspectives from both the villagers and the healthcare providers were obtained and analyzed.
- Baseline health-seeking behaviors data were not available but data integration indicated the improvement in health-seeking behaviors in the malaria endemic district.
- Generalizability might be an issue but the lessons learned might be transferable to other malaria endemic areas in Lao PDR and other similar settings.

For peer review only

For peer review only - http://bmjopen.bmj.com/site/about/guidelines.xhtml

Introduction

Malaria threatens lives.[1] In 2018, 405,000 lives were lost to malaria and 228 million cases were reported.[2] Moreover, 272,000 deaths (67%) occurred in children under 5 years of age.[2] Currently, the effort to rid the world of malaria has regained momentum and an ambitious goal to eradicate malaria by 2050 has been set once again.[3] However, the application of the same, presently available tools and approaches will not help us achieve the goal.[4]

In the Greater Mekong Subregion (GMS), malaria mortality has substantially decreased in the past decade.[5] For example, from October-December 2018, the region reported 25,974 malaria cases, equivalent to a 21% decrease compared to the same period in the previous year.[6] However, malaria elimination efforts in many GMS countries have been hampered by difficulties, such as large movements of people across borders, diverse geographical terrains, and the availability and access to counterfeit antimalarials.[7] Furthermore, these factors have also contributed to the emergence of antimalarial drug resistance.[8]

In the Lao People's Democratic Republic (Lao PDR), which is situated in the GMS, the key risk populations have been identified as ethnic minority groups living in remote forested and mountainous areas, plantation workers, migrant workers, and the soldiers.[9 10] Lao PDR is a linguistically and ethnically diverse nation.[11] Ethnic minorities in Lao PDR

For peer review only - http://bmjopen.bmj.com/site/about/guidelines.xhtml

usually inhabit remote mountainous and forest areas and are usually characterized by extreme poverty.[9] Access to routine healthcare services is also limited for these ethnic minorities due to geographical barriers.[7] These ethnic minorities usually have their own distinct languages and many do not speak Lao.[5 9] As a result, dissemination of health messages is challenging.[9] Moreover, many ethnic minorities are animists with their own unique beliefs towards illness such as evil spirits could cause malaria.[11 12] Consequently, many people from these groups still resort to traditional medicine and religious/animistic rituals for treatment.[12]

The level of knowledge on modern medicine in Lao PDR is still low.[13 14] To complicate matters further, substandard and counterfeit medicines are still available in private pharmacies.[13 15] In the context of malaria, this could hamper the ongoing efforts of malaria elimination, endanger patients' lives, and worsen the drug resistance problem in Lao PDR.[13 15]

To achieve the Sustainable Development Goal 3: Good Health & Wellbeing for All; a key element must be attained: achieving Universal Health Coverage (UHC). To this end, the Lao Government has introduced the National Health Insurance (known by the locals as "*Kor Por Sor*") scheme in 2016. In this scheme, all Lao citizens can receive treatments at public health facilities at a very minimal cost. For example, for outpatient visits at a village health center, villagers pay only 5000 kip (approximately US$ 0.63, as of Jan 2019) per visit. However, the implementation of the National Health Insurance scheme still faces barriers such as low awareness of this scheme among the villagers.[16]

For peer review only - http://bmjopen.bmj.com/site/about/guidelines.xhtml

To accelerate the progress towards malaria elimination by 2030, understanding the level of knowledge of the populations in malaria endemic areas on modern medicine and their health-seeking behaviors is critical to inform the policymakers on the types of interventions and treatment plans and policies to improve healthcare delivery in Lao PDR. The objective of this study was to explore health-seeking behaviors from the perspectives of the villagers and the frontline healthcare workers in a malaria endemic area in Lao PDR.

Methods

Study design and settings

A convergent mixed methods design was used where both quantitative and qualitative data were simultaneously collected and integrated.[17] In this study, quantitative data were collected from the villagers, and qualitative data from both the villagers and the healthcare workers.

This study was conducted in October 2018 in Thapangthong district, Savannakhet province, Lao PDR. This district was chosen because of the high number of reported malaria cases. In 2018, out of 2245 reported malaria positive cases in Savannakhet province, 323 were from Thapangthong district [Data obtained by personal communication with the Center of Malariology, Parasitology and Entomology, Lao PDR]. Two villages (Villages A and B) were chosen after consultation with the District Health Office in Thapangthong district based on the number of highest malaria cases and consultation with the village headmen.

For peer review only - http://bmjopen.bmj.com/site/about/guidelines.xhtml

Thapangthong district comprises 42 villages, and the population as of October 2018 was 42,563, of which 21,157 were female villagers. The district has one district hospital and the ethnic composition of this district is 42% Lao ethnic group and 58% Katang ethnic group. The population of Village A was 1149 villagers living in 215 households. Out of 1149 villagers, 570 were women. The major ethnic group in Village A is Lao. Village B has 753 villagers living in 125 households. Out of 753 villagers, 324 were women. The major ethnic group in Village B is Katang.

Study participants and recruitment

Study participants in this study were villagers and healthcare workers aged 18 years and above living and working in the two villages. After Village A and Village B were chosen, the village headmen of each village were informed the purpose of this study. For quantitative data collection, the village headmen informed the whole village a few days before the study took place, and requested a representative of each household who agreed and were willing to participate in the study to come at the specified time and place (either at the village meeting room or the meeting room of the health center).

For qualitative data collection, villagers who finished the quantitative interview were personally approached and informed about the focus group discussions (FGDs). All the villagers who volunteered were included. For the in-depth interviews (IDIs) of the healthcare workers, all the healthcare workers were approached and informed of the purpose of the

For peer review only - http://bmjopen.bmj.com/site/about/guidelines.xhtml

135 study. All those who agreed to participate were interviewed.

136 **Quantitative part**

137 A structured questionnaire was created by adapting the content from the latest Lao
138 Social Indicator Survey,[18] Malaria Indicator Survey from Roll Back Malaria,[19 20] and clinical
139 vignettes from a study by Mebratie et al.[21] The questionnaire consisted of seven sections:
140 health-seeking behaviors, knowledge on malaria and modern medicine, perceived severity
141 and perceived susceptibility, perceived benefits, self-efficacy, clinical vignettes, and
142 socioeconomic characteristics.

143 The questionnaires were translated into Lao by two authors (P.K. and S. Ke., both
144 medical doctors and malaria researchers) who are Lao native speakers and fluent in English.
145 They also checked each other's translation for accuracy and checked the questionnaire for
146 content and face validity. Before data collection, six research assistants, who were medical
147 doctors or healthcare worker, were trained on the content of the questionnaire and ethical
148 issues such as privacy protection of the participants. The questionnaire was pre-tested in
149 another village in the same district among 14 villagers. After the pretest, a meeting with all
150 the research assistants was held to revise the questionnaires for easier understanding based on
151 their feedbacks.

152 Six research assistants collected the quantitative data by interviewer-administered
153 questionnaire using KoBo Collect application downloaded on password protected Android

For peer review only

tablets provided to each research assistant. Each villager who finished answering the questionnaire was provided with a bag of sanitary items such as soap and washing detergents as an incentive.

Descriptive statistics were computed to summarize the socio-demographic and socio-economic characteristics of the participants. Chi-square test was used for categorical variables. Multiple logistic regressions were used to identify the factors associated with perceived severity and perceived susceptibility. In both models, the explanatory variables were village, sex, ethnicity, malaria experience, and knowing that malaria is caused by mosquito bites. For clinical vignettes, providers such as village health volunteers, health centers, district hospitals, and provincial hospitals were categorized as 'public health facilities.' Other providers were grouped as 'others.' Statistical significance was set at $p<0.05$. All statistical analyses were performed with Stata/IC 13.1 (College Station, Texas, USA). This study is in accordance with the Strengthening the Reporting of Observational studies in Epidemiology (STROBE) Statement checklist (Supplementary 1).[22]

Qualitative part

FGDs were conducted among villagers and IDIs among healthcare workers. Three authors (K.I.C.O., P.K., S.Ke.) collected data until saturation was reached, meaning no new information emerging from the study participants even after additional FGDs or IDIs were conducted.

For peer review only - http://bmjopen.bmj.com/site/about/guidelines.xhtml

FGD and IDI topic guides were created based on the quantitative questionnaire for the villagers. The FGD topic guide prompted the villagers to elaborate their experience with malaria, their choice of treatment, the reasons behind their decisions, health-seeking behaviors in general, and the cause of malaria. As for the healthcare workers, the IDI topic guide asked about the health-seeking behaviors of the villagers, and the barriers in providing proper care.

All FGDs and IDIs were audio recorded and field notes were taken. Each villager and healthcare worker who finished the FGD or IDI was provided with a bag of sanitary items such as soap and washing detergents as an incentive. Audio data were transcribed verbatim in Lao language by native Lao research assistants. All the transcripts were double checked for accuracy. Thematic analysis was used to analyze the data and was performed by three authors (K.I.C.O., P.K., S.Ke.). To ensure trustworthiness in the analysis, the steps outlined by Nowell et al.[23] were followed. NVivo 12 Pro (QRS International Pty. Ltd, Doncaster, Australia) software was used to expedite the data analysis process. Guidelines in the Consolidated Criteria for Reporting Qualitative Research (COREQ) were followed (Supplementary 2).[24]

Data integration

Both the quantitative data and qualitative data were integrated to gain new insights through joint displays. A joint display is a visual means to combine both qualitative and

For peer review only - http://bmjopen.bmj.com/site/about/guidelines.xhtml

192 quantitative data.[25 26]

193 **Ethical considerations**

194 Ethical approval was obtained from the Research Ethics Committee of the Graduate
195 School of Medicine, The University of Tokyo (Serial number: 12038) and the National Ethics
196 Committee for Health Research, Ministry of Health, Lao PDR (No. 094/NECHR).
197 Participation in this study was voluntary and the research assistants explained the purpose of
198 the study prior to the data collection to the villagers and healthcare workers. A written
199 informed consent was obtained from each participant. Personally identifiable information was
200 not collected and the IDIs and FGDs were conducted in a secured place with low risk of
201 being overheard by outsiders, such as the village meeting room and the meeting room of the
202 health center.

203 **Results**

204 **Quantitative part**

205 Table 1 shows the sociodemographic characteristics of the villagers. In total, data
206 were collected from 313 villagers; 202 from Village A and 111 from Village B. The number
207 of female villagers in Village A was 128 (63.4%) while in Village B it was 44 (39.6%). In
208 Village A, 198 (98.0%) villagers were Buddhists while in Village B, 81 (73.0%) were
209 animists. The major ethnic group in Village A was Lao (197, 97.5%) while that of Village B
210 was Katang (89, 80.2%). In both villages, the majority of the villagers were farmers (Village

For peer review only - http://bmjopen.bmj.com/site/about/guidelines.xhtml

A: 170 (84.2%); Village B: 105 (94.6%)).

[INSERT TABLE 1 HERE]

Table 2 shows the first place to seek treatment when a family member falls sick in both villages. Half of the villagers in Village A (101, 50.0%) would go to the health center first compared to 105 (94.6%) villagers in Village B.

[INSERT TABLE 2 HERE]

Table 3 shows the results of knowledge of malaria and treatment among the villagers. In Village A, 115 (56.9%) villagers answered that malaria is caused by mosquito bites compared to 85 (76.6%) villagers in Village B. Drinking dirty water was mentioned as a cause of malaria by 30 (14.9%) villagers in Village A and 27 (24.3%) villagers in Village B. In both villages, no villagers attributed witchcraft to be the cause of malaria. Regarding protection against malaria, 150 (74.3%) villagers in Village A answered sleeping under an insecticide-treated bed net compared to 101 (91.0%) villagers in Village B. In Village A, 23 (11.4%) villagers thought that not drinking dirty water could protect oneself from malaria compared to 28 (25.2%) villagers in Village B. Most villagers in both villages did not know the most effective treatment for malaria (Village A: 185 (91.6%); Village B: 85 (76.6%)).

[INSERT TABLE 3 HERE]

Table 4 shows the individual beliefs of the villagers in the context of malaria. In Village A, 109 (54.0%) villagers perceived that the consequences of malaria were serious

For peer review only - http://bmjopen.bmj.com/site/about/guidelines.xhtml

For peer review only

compared to 60 (54.1%) villagers in Village B. Regarding perceived susceptibility, 112 (55.5%) villagers in Village A perceived that they were at high risk of malaria compared to 61 (55.0%) in Village B. As for perceived benefits, bed net use was thought to reduce malaria risk by 126 (62.4%) villagers in Village A compared to 71 (64.0%) villagers in Village B.

[INSERT TABLE 4 HERE]

Table 5 shows the responses of the villagers to clinical vignettes describing five conditions: hemolytic anemia, malaria, tuberculosis, acute respiratory infection, and diarrhea. For malaria, 194 (96.0%) villagers in Village A responded that they would first seek treatment at public health facilities while 109 (98.2%) villagers in Village B responded that they would do the same. In Village B, 97-98% of the villagers answered that they would first seek treatment at the public health facilities for other conditions.

[INSERT TABLE 5 HERE]

Table 6 shows the factors associated with both perceived severity of malaria and perceived susceptibility of malaria. For perceived severity of malaria, those who have not experienced malaria before were more likely to perceive that the consequences of malaria were serious compared to those who have experienced malaria before (AOR=1.69, 95% CI: 1.03 to 2.75). Village, sex, ethnicity, malaria experience, and knowing about the cause of malaria were not significantly associated with perceived susceptibility.

[INSERT TABLE 6 HERE]

For peer review only - http://bmjopen.bmj.com/site/about/guidelines.xhtml

Qualitative part

In Village A, two male and two female FGDs were conducted while in Village B, three FGDs each for male and female villagers were conducted. The number of villagers per group was 3-4. Supplementaries 3 and 4 show the characteristics of the villagers who participated in the FGDs and healthcare workers who participated in the IDIs. The FGDs and IDIs lasted about an hour. Supplementary 5 shows the themes that emerged from the FGDs among the villagers.

Theme: Attributing the cause of malaria to something else other than being bitten by mosquitoes.

Some villagers believed that malaria was cause by something else other than mosquito bites. For example, some villagers mentioned ghosts or unclean water as the cause of malaria.

"When I had malaria, they used 'pee pop' (ghost) to exorcize it away." (Village A, female, 30s, farmer)

"Drinking unclean water, you will get malaria." (Village B, male, 10s, farmer)

Theme: Attributing the cause of malaria to being bitten by mosquitoes.

In contrast with the previous theme, some villagers correctly attributed the cause of malaria to being bitten by mosquitoes.

"*Because of mosquito bites. When you do not sleep under the bed net, the mosquitoes will bite you.*" (Village B, male, 20s, government servant)

For peer review only - http://bmjopen.bmj.com/site/about/guidelines.xhtml

Theme: Describing an illness using local terms.

The villagers also described common illnesses in the area using local terms and local languages.

"'Kai luad niao' (sticky blood fever), when you are dehydrated, your blood will be sticky. When you drink water, your blood will dilute." (Village A, female, 40s, farmer)

"'Kai nyoong' (literally translated from Lao as 'mosquito fever', usually referred to malaria) in Katang language is called 'ae muay'" (Village B, female, 40s, farmer)

Theme: Expressing hope to improve the health center.

In the FGDs, the villagers expressed their hopes that the health center would be improved.

"I hope the staff will be more knowledgeable on the treatments." (Village B, male, 30s, farmer)

"I hope the village health center will be cleaner. There are mosquitoes here at the health center too, so you might even get malaria here!" (Village B, female, 20s, farmer)

Theme: Expressing difficulties in getting health services.

The villagers also mentioned about their difficulties in getting health services. Some of the difficulties mentioned were financial or road conditions. In the following quote, the male villager used a local saying to illustrate that lack of financial resources could hinder one from getting medical services.

"'Kon luai bor dai kau kuk, kon tuk bor dai kau hong mor' (The rich do not go to jail, the

For peer review only - http://bmjopen.bmj.com/site/about/guidelines.xhtml

poor do not go to the hospital)" (Village B, male, 20s, farmer)

"I hope the roads will also be improved. When it is raining, people die because they are not sent to the hospital in time." (Village B, male, 30s, farmer)

Theme: Recalling personal experience

Although the district has one district hospital and village health centers, these facilities were not equipped to handle complicated cases and the patients must be referred to the provincial hospital, which is a 5- to 7-hour drive in the dry season and longer in the rainy season.

"*We do not have oxygen here in the district hospital. I gave birth to a premature baby once and it died because we did not have oxygen. We were going to the Savannakhet provincial hospital and the baby died on the way in Pakxong. No incubator too.*" (Village A, female, 30s, businesswoman)

Theme: Mentioning about the National Health Insurance ("*Kor Por Sor*").

However, the villagers mentioned that the recently introduced National Health Insurance ("*Kor Por Sor*") was a facilitator in seeking treatment from the health center.

"I will go to the health center first, because I have to pay only 5000 kip. If I go to a private clinic, I will have to pay at least 50000 kip" (Village A, female, 30s, farmer)

Three IDIs were conducted in Village A (two for healthcare workers and one for village health volunteer) and four were conducted in Village B (three for healthcare workers

For peer review only - http://bmjopen.bmj.com/site/about/guidelines.xhtml

For peer review only

and one for village health volunteer). The major themes were "Mentioning about the National Health Insurance *("Kor Por Sor")*" and "Expressing hope to improve the health center." Supplementary 6 shows all other themes that emerged during the IDIs with the healthcare workers.

Theme: Mentioning about the National Health Insurance ("*Kor Por Sor*").

When asked why the villagers come to the health centers, the healthcare workers also mentioned the National Health Insurance ("*Kor Por Sor*") as the facilitator.

"Now there is the 'Kor Por Sor' (National Health Insurance) system, where you have to pay only 5000 kip, they will come even when they have common cold." (Village A, female 30s)

"Because of 'Kor Por Sor' (National Health Insurance), more people come to seek treatment at this health center." (Village B, male, 30s)

Theme: Expressing hope to improve the health center.

The healthcare workers also mentioned about their hopes to improve the health center and also improve their skills and knowledge to better serve the people.

"The knowledge and capability. I want to learn more. Another thing is the building. Too small. When people are sick they can't sleep here. If possible, I hope for more advanced equipment too." (Village B, male, 30s)

"At this health center the manpower is not enough, we also do not have enough knowledge. I hope people with knowledge will come here to work at this health center. I also hope this

For peer review only

health center will be expanded. It is very crowded when there are many sick people and people who give birth." (Village B, female, 20s)

Data integration

Figures 1-3 show the joint displays combining both quantitative and qualitative data. Although in both villages, more than half of the villagers knew that malaria is caused by mosquito bites, some villagers still attributed the cause of malaria to drinking dirty water or water contaminated with mosquito larvae (Figure 1).

When probed about the first place to seek treatment when a family member was sick, 72.8% from Village A and 98.2% from Village B indicated that they would go to either the health center or district hospital. In the context of malaria, a vast majority, 96.0% in Village A and 98.2% in Village B answered that they would first seek treatment at the public health facilities. In the qualitative part, both villagers and healthcare workers mentioned that after the introduction of the National Health Insurance ("*Kor Por Sor*"), the treatment cost became cheaper and this was the facilitator for the villagers to seek treatment at the health center or the district hospital (Figure 2).

Although most of the villagers seek treatment from the health center or the district hospital, and are confident to seek treatment when having malaria (98.0% from Village A and 96.4% from Village B), many problems and dissatisfactions exist. For example, the lack of medication and proper equipment and the attitude of the healthcare workers might discourage

For peer review only - http://bmjopen.bmj.com/site/about/guidelines.xhtml

the villagers from seeking proper healthcare treatment (Figure 3).

Discussion

This study has three major findings. First, in the quantitative part, most of the villagers in both villages would seek treatment at the health center or the district hospital both in the context of malaria and other common diseases in the area. The level of knowledge of the villagers on the cause of malaria was low and the tendency to link malaria to causes other than mosquito bites was common. Second, in the qualitative part, both villagers and healthcare workers attributed increase in the number of villagers seeking treatment at the health center or the district hospital to the introduction of the National Health Insurance ("*Kor Por Sor*"). However, FGDs and IDIs revealed problems faced by the villagers in seeking treatment and healthcare workers in providing care. Third, even though the introduction of the National Health Insurance ("*Kor Por Sor*") encouraged many villagers to seek treatment from the health center or the district hospital, both villagers and the healthcare workers were still facing hurdles in getting and providing optimal care. Although the two villages vary in terms of ethnic groups and religions, the health-seeking behaviors and the barriers they faced in receiving optimal healthcare services were similar.

In this study, villagers from both villages mainly sought treatment from the public healthcare providers: village health volunteer, health center, or the district hospital. In Lao PDR, health-seeking behaviors are influenced by several factors such as distance to a health

For peer review only - http://bmjopen.bmj.com/site/about/guidelines.xhtml

center, socioeconomic factors, and family or friends' influence.[27 28] Compared to another study in Nong District, Savannakhet Province,[28] the number of villagers seeking treatment from traditional healers in this study was very low to none. The high usage of village public healthcare providers in this study might be due to the ease in accessing these public healthcare providers as the health centers of both villages were situated in the villages.[29] Moreover, in another study in Lao PDR, people who initially visited a public healthcare provider were more likely to maintain a connection with the provider.[30]

In the FGDs of the villagers and IDIs with the healthcare workers, one of the major reasons that increased the number of villagers seeking treatment at the health center or the district hospital was the introduction of the National Health Insurance ("*Kor Por Sor*"). In this scheme, villagers pay only 5000 kip (approximately US$ 0.63, as of Jan 2019) to get basic treatment and medication at the health center and 10000 kip (approximately US$ 1.25, as of Jan 2019) at the district hospital.[16] Patients only need to bring the family registration book to prove their residence in the village. For those households without the family registration book, a special certificate issued by the village head is also acceptable [Personal communication with a Lao counterpart, based on an official Ministerial Order document]. In Lao PDR, although malaria diagnosis and treatment are free, patients had to pay for treatment of other illnesses and those from the low-income bracket could not afford the treatment.[31] As economic status is a major barrier to getting proper treatment, the National Health Insurance

For peer review only - http://bmjopen.bmj.com/site/about/guidelines.xhtml

has made it more affordable and accessible for the villagers to get treatment at the health center or the district hospital.

In this study, many villagers still attributed the cause of malaria to factors other than being bitten by mosquitoes, such as drinking unclean water. This result is consistent with another study among military personnel in Lao PDR.[31] Having knowledge on the cause of malaria does not necessarily lead to protective actions, but having knowledge can be a facilitator for self-implemented protective measures against malaria.[32] In the FGDs in both villages, many villagers described malaria and symptoms using local terms. This result emphasizes the need to tailor health messages and elimination strategies that are locally acceptable and understandable.[12 33]

In addition, villagers who have not experienced malaria before in this study were more likely to perceive malaria to be more severe than those who experienced before. Asymptomatic malaria carriers are very common in endemic areas in Lao PDR and these carriers do not exhibit the usual malaria symptoms due to repeated infections.[34 35] Thapangthong district was no exception and those who had experienced malaria before could be asymptomatic carriers. This result also highlights the necessity to tackle asymptomatic infections in Lao PDR in order to achieve the elimination goal.

Although the quantitative results showed high usage of health centers and the district hospital, both the FGDs with the villagers and IDIs with the healthcare workers revealed

For peer review only - http://bmjopen.bmj.com/site/about/guidelines.xhtml

many barriers in treatment seeking and treatment delivering in the district. In this study, unavailability of medications and the lack of proper medical equipment were some of the major grievances of the villagers. Moreover, the current reporting system for malaria cases and antimalarial stocks at the village level to the district level in many malaria endemic areas in Lao PDR is still paper-based. This could cause a logistical delay in delivering the antimalarial stocks from Vientiane Capital to the endemic areas. In addition, some villagers also mentioned the unpleasant experience due to the attitude of the healthcare providers. Moreover, some of the healthcare providers acknowledged that they were not skilled enough in providing all treatments and hoped for better training. Lao PDR still faces many challenges in building a high-quality and equitable primary healthcare centers throughout the country.[27] The major challenges are difficult geographical terrains, inadequate training of healthcare staffs, lack of qualified health workers, low salaries, and poor morale among the staff.[27 36] Therefore, although the current usage of the health center and the district hospital is high, it is crucial to strengthen the capacity of the frontline healthcare facilities to monitor and eliminate malaria and improve the reporting mechanism by introducing online reporting system, to improve the overall health outcomes in the country.

This study has several strengths. This study provided evidence of the positive effect of the National Health Insurance ("*Kor Por Sor*"). Moreover, by using mixed methods where quantitative data and qualitative data were collected and interpreted together, this study

For peer review only - http://bmjopen.bmj.com/site/about/guidelines.xhtml

provided a more comprehensive picture of the health-seeking behavior situation in Lao PDR, compared to using quantitative data or qualitative data alone.

However, the results of this study must be interpreted considering several limitations. First, in the FGDs and IDIs, social desirability might have affected the responses of both the villagers and healthcare workers. However, to obtain an honest response and to increase the credibility of the data, the researchers have established a good working relationship with the villagers and healthcare workers. Second, the results of this study are not representative of all malaria endemic districts of Lao PDR with different ethnic groups and different geographical terrains. However, transferability of the lessons learned from this study is possible by focusing on the key common issues affecting other malaria endemic areas such as lack of health personnel and the need to improve the healthcare facilities.

Conclusions

Although the treatment seeking in public healthcare facilities in this study was high, the knowledge regarding malaria transmission was low. Moreover, both the villagers and healthcare providers expressed dissatisfaction with and the desire to improve the quality of the public healthcare facilities in this study. The introduction of the National Health Insurance ("*Kor Por Sor*") was regarded positively by both the villagers and the healthcare workers and this is a first step to achieving universal health coverage in Lao PDR. Therefore, a more integrated approach to improve healthcare seeking and healthcare delivery focusing

For peer review only - http://bmjopen.bmj.com/site/about/guidelines.xhtml

on the villagers, healthcare providers, and the facilities should be accelerated.

Acknowledgements We would like to thank Dr. Chanthalangsouk Soukhavady, Dr. Syda Keothavone, Dr. Souphaphone Phimmasan, and Ms. Chansamone Khongkham for assisting in the data collection. Our gratitude also goes to the dedicated staff at the Center of Malaria Parasitology and Entomology, Savannakhet Provincial Health Office, and Thapangthong District Health Office for their support. We would also like to thank the villagers and healthcare workers who participated in this study.

Contributors KICO conceived and designed this study with the supervision from MJ. KICO, PK, SKe collected and analyzed the data. MI, PTB, SKa, and MJ provided critical comments and support during data collection and analysis. KICO wrote the first draft of the manuscript. All authors read and approved the final version of the manuscript.

Funding This study was financially supported by the JICA/AMED SATREPS project for the Development of innovative research techniques in the genetic epidemiology of malaria and other parasitic diseases in the Lao PDR for containing their expanding endemicity. The funder had no role in designing the study, collecting, analyzing and interpreting the data, preparing the manuscript, or decision to publish.

Competing interests None declared

For peer review only - http://bmjopen.bmj.com/site/about/guidelines.xhtml

458 **Patient consent for publication** Not required

459 **Patient and public involvement** No patients or members of the public were involved in the
460 design, implementation, analyses, or reporting of the research.

461 **Ethics approval** This study was approved by the National Ethics Committee for Health
462 Research (NECHR), National Institute of Public Health (NIOPH), Ministry of Health, Lao
463 PDR (No. 049 NIOPH/NECHR) and the Research Ethics Committee of the University of
464 Tokyo (No. 12038). Participation was voluntary and a written informed consent was obtained
465 from all participants.

466 **Data availability statement** Data are available upon reasonable request to the corresponding
467 author.

468

469 Figure 1 Joint display of knowledge of the cause of malaria

470 Figure 2 Joint display of the impact of National Health Insurance

471 Figure 3 Joint display of the barriers in treatment seeking

472

473 **References**

474 1. World Health Organization. Malaria. [https://www.who.int/en/news-room/fact-
475 sheets/detail/malaria] Accessed 10 September 2019.
476 2. World Health Organization. World Malaria Report 2019. Geneva; 2019.
477 3. Feachem RGA, Chen I, Akbari O, Bertozzi-Villa A, Bhatt S, Binka F, et al. Malaria
478 eradication within a generation: ambitious, achievable, and necessary. Lancet. 2019, 394:1056-
479 1112.

For peer review only - http://bmjopen.bmj.com/site/about/guidelines.xhtml

4. Ghebreyesus TA. The malaria eradication challenge. Lancet. 2019, 394:990-991.

5. Hewitt S, Delacollette C, Chavez I. Malaria situation in the Greater Mekong Subregion. Southeast Asian J Trop Med Public Health. 2013, 44 Suppl 1:46-72; discussion 306-307.

6. World Health Organization. Mekong Malaria Elimination Programme Epidemiology Summary Volume 4, October-December 2018. Geneva; 2018.

7. Cui L, Yan G, Sattabongkot J, Cao Y, Chen B, Chen X, et al. Malaria in the Greater Mekong Subregion: heterogeneity and complexity. Acta Trop. 2012, 121:227-239.

8. Packard RM. The origins of antimalarial-drug resistance. N Engl J Med. 2014, 371:397-399.

9. Ministry of Health of the Lao People's Democratic Republic. National Strategy for Malaria Control and Elimination 2015-2020. Vientiane; 2015.

10. World Health Organization. Strategy for malaria elimination in the Greater Mekong Subregion (2015-2030). Geneva; 2015.

11. Messerli P, Heinimann A, Epprecht M, Phonesaly S, Thiraka C, Minot N. Socio-Economic Atlas of the Lao PDR an Analysis based on the 2005 Population and Housing Census. Swiss National Center of Competence in Research (NCCR) North-South and Geographica Bernensia; 2008.

12. Shirayama Y, Phompida S, Kuroiwa C. Modern medicine and indigenous health beliefs: malaria control alongside "Sadsana-phee" (animist belief system) in Lao PDR. Southeast Asian J Trop Med Public Health. 2006, 37:622-629.

13. Caillet C, Sichanh C, Syhakhang L, Delpierre C, Manithip C, Mayxay M, et al. Population awareness of risks related to medicinal product use in Vientiane Capital, Lao PDR: a cross-sectional study for public health improvement in low and middle income countries. BMC Public Health. 2015, 15:590.

14. Alvesson HM, Lindelow M, Khanthaphat B, Laflamme L. Coping with uncertainty during healthcare-seeking in Lao PDR. BMC Int Health Hum Rights. 2013, 13:28.

15. Tabernero P, Mayxay M, Culzoni MJ, Dwivedi P, Swamidoss I, Allan EL, et al. A Repeat Random Survey of the Prevalence of Falsified and Substandard Antimalarials in the Lao PDR: A Change for the Better. Am J Trop Med Hyg. 2015, 92:95-104.

16. World Health Organization. Lao Ministry of Health moves forward with National Health Insurance. [https://www.who.int/laos/news/detail/01-09-2019-lao-ministry-of-health-moves-forward-with-national-health-insurance] Accessed 5 December 2019.

17. Creswell JW. A concise introduction to mixed methods research. Los Angeles: Sage; 2015.

18. Ministry of Health and Lao Statistics Bureau. Lao social indicator survey 2011-12 (multiple indicator cluster survey/demographic and health survey). Vientiane: Ministry of Health and Lao Statistics Bureau; 2012.

19. RBM Partnership. Malaria Behavior Change Communication (BCC) Indicator

For peer review only - http://bmjopen.bmj.com/site/about/guidelines.xhtml

Reference Guide. Geneva; 2014.
20. RBM Partnership. Malaria Social and Behavior Change Communication Indicator Reference Guide. 2nd ed. Geneva; 2017.
21. Mebratie AD, Van de Poel E, Yilma Z, Abebaw D, Alemu G, Bedi AS. Healthcare-seeking behaviour in rural Ethiopia: evidence from clinical vignettes. BMJ Open. 2014, 4:e004020.
22. von Elm E, Altman DG, Egger M, Pocock SJ, Gøtzsche PC, Vandenbroucke JP, et al. The Strengthening the Reporting of Observational Studies in Epidemiology (STROBE) statement: guidelines for reporting observational studies. PLoS Med. 2007, 4:e296.
23. Nowell LS, Norris JM, White DE, Moules NJ. Thematic Analysis: Striving to Meet the Trustworthiness Criteria. Int J Qual Methods. 2017, 16.
24. Tong A, Sainsbury P, Craig J. Consolidated criteria for reporting qualitative research (COREQ): a 32-item checklist for interviews and focus groups. Int J Qual Health Care. 2007, 19:349-357.
25. Fetters MD, Curry LA, Creswell JW. Achieving Integration in Mixed Methods Designs-Principles and Practices. Health Serv Res. 2013, 48:2134-2156.
26. Guetterman TC, Fetters MD, Creswell JW. Integrating Quantitative and Qualitative Results in Health Science Mixed Methods Research Through Joint Displays. Ann Fam Med. 2015, 13:554-561.
27. Bochaton A. Cross-border mobility and social networks: Laotians seeking medical treatment along the Thai border. Soc Sci Med. 2015, 124:364-373.
28. Adhikari B, Phommasone K, Pongvongsa T, Koummarasy P, Soundala X, Henriques G, et al. Treatment-seeking behaviour for febrile illnesses and its implications for malaria control and elimination in Savannakhet Province, Lao PDR (Laos): a mixed method study. BMC Health Serv Res. 2019, 19:252.
29. Suswardany DL, Sibbritt DW, Supardi S, Chang S, Adams J. A critical review of traditional medicine and traditional healer use for malaria and among people in malaria-endemic areas: contemporary research in low to middle-income Asia-Pacific countries. Malar J. 2015, 14:98.
30. Nonaka D, Vongseththa K, Kobayashi J, Bounyadeth S, Kano S, Phompida S, et al. Public and private sector treatment of malaria in Lao PDR. Acta Trop. 2009, 112:283-287.
31. Vilay P, Nonaka D, Senamonty P, Lao M, Iwagami M, Kobayashi J, et al. Malaria prevalence, knowledge, perception, preventive and treatment behavior among military in Champasak and Attapeu provinces, Lao PDR: a mixed methods study. Trop Med Health. 2019, 47.
32. Matsumoto-Takahashi EL, Tongol-Rivera P, Villacorte EA, Angluben RU, Yasuoka J, Kano S, et al. Determining the impact of community awareness-raising activities on the prevention of malaria transmission in Palawan, the Philippines. Parasitol Int. 2014, 63:519-

For peer review only - http://bmjopen.bmj.com/site/about/guidelines.xhtml

526.
33. Nofal SD, Peto TJ, Adhikari B, Tripura R, Callery J, Bui TM, et al. How can interventions that target forest-goers be tailored to accelerate malaria elimination in the Greater Mekong Subregion? A systematic review of the qualitative literature. Malar J. 2019, 18:32.
34. Pongvongsa T, Nonaka D, Iwagami M, Nakatsu M, Phongmany P, Nishimoto F, et al. Household clustering of asymptomatic malaria infections in Xepon district, Savannakhet province, Lao PDR. Malar J. 2016, 15:508.
35. Iwagami M, Keomalaphet S, Khattignavong P, Soundala P, Lorphachan L, Matsumoto-Takahashi E, et al. The detection of cryptic Plasmodium infection among villagers in Attapeu province, Lao PDR. PLoS Negl Trop Dis. 2017, 11:e0006148.
36. Kounnavong S, Gopinath D, Hongvanthong B, Khamkong C, Sichanthongthip O. Malaria elimination in Lao PDR: the challenges associated with population mobility. Infect Dis Poverty. 2017, 6:81.

For peer review only

For peer review only - http://bmjopen.bmj.com/site/about/guidelines.xhtml

Table 1 Sociodemographic characteristics of the villagers

	Village A	Village B
Villagers interviewed	202	111
Sex, n (%)		
Male	74 (36.6)	67 (60.4)
Female	128 (63.4)	44 (39.6)
Age		
Minimum	18	18
Maximum	85	73
Median	40	39
Mean	40.9	38.6
Religion, n (%)		
Buddhism	198 (98.0)	30 (27.0)
Animism	4 (2.0)	81 (73.0)
Ethnicity, n (%)		
Lao	197 (97.5)	21 (18.9)
Katang	2 (1.0)	89 (80.2)
Do not know	3 (1.5)	1 (0.9)
Marital status, n (%)		
Married	183 (90.6)	97 (87.4)
Single	5 (2.5)	6 (5.4)
Divorced	2 (1.0)	1 (0.9)
Widowed	12 (5.9)	6 (5.4)
Others	0	1 (0.9)
Occupation, n (%)		
Farmer	170 (84.2)	105 (94.6)
Soldier	1 (0.5)	0
Housewife	18 (8.9)	2 (1.8)
Businessman/women	1 (0.5)	1 (0.9)
Government servant	7 (3.5)	2 (1.8)

For peer review only

For peer review only - http://bmjopen.bmj.com/site/about/guidelines.xhtml

Table 1 Sociodemographic characteristics of the villagers (continued)

	Village A	Village B
Others	5 (2.5)	1 (0.9)
Education, n (%)		
No education	71 (35.2)	42 (37.8)
Preschool	1 (0.5)	1 (0.9)
Primary school	84 (41.6)	46 (41.4)
Lower secondary school	28 (13.9)	14 (12.6)
Upper secondary school	8 (4.0)	5 (4.5)
Post secondary non-tertiary	8 (4.0)	2 (1.8)
Tertiary	2 (1.0)	1 (0.9)
Average monthly household income (kip) (US$1=8000 kip [Jan 2019])		
Minimum	0	0
Maximum	10000000	4000000
Median	200000	100000
Mean	496881	290000
Number of household members (Median)	5	6
Have insecticide-treated nets at home		
Yes	169 (83.7)	107 (96.4)
No	31 (15.4)	4 (3.6)
Don't know	2 (1.0)	0

**Percentage may not add up to 100 due to rounding*

572

573

For peer review only - http://bmjopen.bmj.com/site/about/guidelines.xhtml

Table 2 First place to seek treatment when a family member falls sick

Where to seek treatment, n (%)	Village A (n=202)	Village B (n=111)
Village health volunteer	25 (12.4)	0
Health center	101 (50.0)	105 (94.6)
District hospital	46 (22.8)	4 (3.6)
Provincial hospital	6 (3.0)	1 (0.9)
Private pharmacy	10 (5.0)	0
Traditional healer	1 (0.5)	0
Others	13 (6.4)	1 (0.9)

For peer review only

For peer review only - http://bmjopen.bmj.com/site/about/guidelines.xhtml

Table 3 Knowledge of malaria and treatment

	Village A (n=202)	Village B (n=111)
What causes malaria? n (%)		
Mosquito bites	115 (56.9)	85 (76.6)
Eating dirty food	17 (8.4)	9 (8.1)
Eating uncooked food	6 (3.0)	7 (6.3)
Drinking dirty water	30 (14.9)	27 (24.3)
Witchcraft	0	0
Going to the forest	73 (36.1)	49 (44.1)
Changing weather	8 (4.0)	3 (2.7)
Getting soaked with rain	3 (1.5)	1 (0.9)
How to protect yourself against malaria? n (%)		
Sleep under an insecticide-treated mosquito net	150 (74.3)	101 (91.0)
Use mosquito repellent	9 (4.5)	4 (3.6)
Avoid mosquito bites	9 (4.5)	5 (4.5)
Avoid going to the forest	10 (5.0)	4 (3.6)
Take preventive medicine	2 (1.0)	0
Spray house with insecticide	8 (4.0)	1 (0.9)
Use mosquito coils	10 (5.0)	4 (3.6)
Fill in puddles around the house	18 (8.9)	3 (2.7)
Keep surrounding of the house clean	54 (26.7)	42 (37.8)
Burn leaves	4 (2.0)	10 (9.0)
Do not drink dirty water	23 (11.4)	28 (25.2)
Do not eat dirty food	8 (4.0)	12 (10.8)
Put mosquito screens on the window	0	1 (0.9)
Do not get soaked with rain water	0	0
What are the symptoms that indicate a person has malaria? n (%)		
Fever	115 (56.9)	70 (63.1)
Feeling cold	89 (44.1)	73 (65.8)
Headache	116 (57.4)	85 (76.6)
Nausea and vomiting	30 (14.9)	17 (15.3)
Diarrhea	2 (1.0)	2 (1.8)
Dizziness	16 (7.9)	13 (11.7)

For peer review only - http://bmjopen.bmj.com/site/about/guidelines.xhtml

Loss of appetite	12 (5.9)	4 (3.6)
Bodyache/Joint pain	58 (28.7)	23 (20.7)
Pale eyes	5 (2.5)	0
Feeling weak	14 (6.9)	9 (8.1)
What is the most effective medication to treat malaria? n (%)		
Chloroquine	0	1 (0.9)
Quinine	2 (1.0)	3 (2.7)
Artemisinin combination therapy (Coartem TM)	3 (1.5)	12 (10.8)
Aspirin, panadol, paracetamol	12 (5.9)	8 (7.2)
Do not know	185 (91.6)	85 (76.6)
What are the main danger signs of malaria? n (%)		
Seizure	29 (14.4)	16 (14.4)
Fainting	7 (3.5)	5 (4.5)
Any fever	8 (4.0)	1 (0.9)
High fever	26 (12.9)	20 (18.0)
Stiff neck	2 (1.0)	3 (2.7)
Feeling weak	8 (4.0)	3 (2.7)
Not active	2 (1.0)	2 (1.8)
Chills/shivering	30 (14.9)	24 (21.6)
Not able to eat	4 (2.0)	1 (0.9)
Vomiting	9 (4.5)	18 (16.2)
Crying all the time	4 (2.0)	1 (0.9)
Restless	19 (9.4)	14 (12.6)
Diarrhea	0	2 (1.8)

For peer review only - http://bmjopen.bmj.com/site/about/guidelines.xhtml

Table 4 Individual beliefs

		Village A (n=202)	Village B (n=111)	p-value
Villagers who perceived the consequences of malaria were serious (Perceived severity) n (%)		109 (54.0)	60 (54.1)	0.987
Villagers who perceived they were at high risk of malaria (Perceived susceptibility) n (%)		112 (55.5)	61(55.0)	0.933
Villagers who believed that the recommended practice or product will reduce their risk (Perceived benefits) n (%)				
	Bed net use	126 (62.4)	71 (64.0)	0.781
	Malaria diagnostics	120 (59.4)	64 (57.7)	0.764
	Malaria treatments with ACT	159 (78.7)	88 (79.3)	0.906
Villagers who are confident in their abilities to perform a specific malaria-related behavior (Self-efficacy) n (%)				
	Protection of self and family	158 (78.2)	76 (68.5)	0.057
	Bed net use	198 (98.0)	110 (99.1)	0.466
	Malaria detection	153 (75.7)	81 (73.0)	0.589
	Seek diagnosis	185 (91.6)	96 (86.5)	0.154
	Seek treatment	198 (98.0)	107 (96.4)	0.384

Chi-square test; ACT=Artemisinin combination therapy

For peer review only - http://bmjopen.bmj.com/site/about/guidelines.xhtml

Table 5 Clinical vignettes: First place to seek treatment

	Hemolytic anemia (n (%))		Malaria (n (%))		Tuberculosis (n (%))		Acute respiratory infection (n (%))		Diarrhea (n (%))	
	Village A (n=202)	Village B (n=111)	Village A (n=202)	Village B (n=111)	Village A (n=202)	Village B (n=111)	Village A (n=202)	Village B (n=111)	Village A (n=202)	Village B (n=111)
Public health facilities	190 (94.1)	109 (98.2)	194 (96.0)	109 (98.2)	189 (93.6)	108 (97.3)	181 (89.6)	109 (98.2)	188 (93.1)	108 (97.3)
Others	9 (4.5)	1 (0.9)	7 (3.5)	1 (0.9)	11 (5.5)	2 (1.8)	20 (9.9)	1 (0.9)	12 (5.9)	2 (1.8)
Take no action	3 (1.5)	1 (0.9)	1 (0.5)	1 (0.9)	2 (1.0)	1 (0.9)	1 (0.5)	1 (0.9)	2 (1.0)	1 (0.9)

For peer review only

For peer review only - http://bmjopen.bmj.com/site/about/guidelines.xhtml

Table 6 Factors associated with perceived severity and perceived susceptibility

Perceived severity of malaria

Characteristics		Adjusted Odds Ratio (95% CI)	p-value
Village			
	Village A	1.00	0.811
	Village B	1.11 (0.48-2.58)	
Sex			
	Male	1.00	0.090
	Female	0.65 (0.40-1.07)	
Ethnicity			
	Lao	1.00	0.615
	Others	0.80 (0.34-1.89)	
Experienced malaria before			
	Yes	1.00	**0.036**
	No	1.69 (1.03-2.75)	
Knowing that malaria is caused by mosquito bites			
	Yes	1.00	0.217
	No	0.74 (0.46-1.20)	

Perceived susceptibility of malaria

Characteristics		Adjusted Odds Ratio (95% CI)	p-value
Village			
	Village A	1.00	0.805
	Village B	0.90 (0.39-2.07)	
Sex			
	Male	1.00	0.910
	Female	1.03 (0.63-1.67)	
Ethnicity			
	Lao	1.00	0.754
	Others	1.14 (0.49-2.67)	
Experienced malaria before			
	Yes	1.00	0.344
	No	1.26 (0.78-2.04)	
Knowing that malaria is caused by mosquito bites			
	Yes	1.00	0.611
	No	0.88 (0.55-1.43)	

For peer review only

Figure 1 Joint display of knowledge of the cause of malaria

**Quantitative Constructs**	**Qualitative Constructs**
56.9% in Village A and 76.6% in Village B attributed the cause of malaria to mosquito bites.<br><br>14.9% in Village A and 24.3% in Village B attributed the cause of malaria to drinking dirty water.<br><br>*(From Table 3 Knowledge of malaria and treatment)*	Theme: Attributing the cause of malaria to something else other than being bitten by mosquitoes.<br><br>*"Because your house is not clean. If you drink water with mosquito eggs, you will get fever."* Village A, female, 40s, government servant.<br><br>*"Drinking unclean water, you will get malaria."* Village B, male, 10s, farmer.
**Comments (meta-inferences)**<br>Many villagers still attributed the main cause of malaria to drinking dirty water or water contaminated with mosquito larvae. Despite efforts by so many stakeholders such as the WHO to eliminate malaria, misconception about the cause of malaria among the villagers can greatly hamper the progress in malaria elimination.	

For peer review only

Figure 2 Joint display of the impact of National Health Insurance

Quantitative Constructs	Qualitative Constructs
72.8% in Village A and 98.2% in Village B would first seek treatment at the health center or district hospital when someone in the family was sick.<br><br>*(From Table 2 First place to seek treatment when a family member falls sick)*<br><br>Responses to the malaria clinical vignette indicated that 96.0% in Village A and 98.2% in Village B would first seek treatment at the public health facilities.<br><br>*(From Table 5 Clinical vignettes)*	Theme: Mentioning about the National Health Insurance ("*Kor Por Sor*")<br><br>*"I will go to the health center first, because I have to pay only 5000 kip. If I go to a private clinic, I will have to pay at least 50000 kip."* Village A, female, 30s, farmer.<br><br>*"Because of 'Kor Por Sor' (National Health Insurance), we have more patients who come here for treatment."* Village B, female, 30s, healthcare worker.
**Comments (meta-inferences)**<br>The recent introduction of the National Health Insurance ("*Kor Por Sor*") seemed to have a positive impact as the financial burden on the villagers is greatly reduced.	

For peer review only

Figure 3 Joint display of the barriers in treatment seeking

Quantitative Constructs	Qualitative Constructs
72.8% in Village A and 98.2% in Village B would first seek treatment in the health center or district hospital when someone in the family was sick.<br><br>*(From Table 2 First place to seek treatment when a family member falls sick)*<br><br>91.6% from Village A and 86.5% from Village B indicated that they were confident to seek malaria diagnosis.<br><br>98.0% from Village A and 96.4% from Village B indicated that they were confident to seek malaria treatment.<br><br>*(From Table 4 Individual beliefs)*	Theme: Expressing opinion on the health center<br>*"Overall everything is good but only one thing is that medicine is always not enough."* Village A, male, 40s, teacher.<br><br>Theme: Recalling personal experience<br>*"We do not have oxygen here in the district hospital. I gave birth to a premature baby once and it died because we did not have oxygen. We were going to the Savannakhet provincial hospital and the baby died on the way in Pakxong. No incubator too."* Village A, female, 30s, businesswoman.
**Comments (meta-inferences)**<br>The ill-equipped facilities are a letdown as prescriptions are not always available and complicated cases will be referred to the provincial hospital which is a 5- to 7-hour drive away in the dry season, and longer in the rainy season. These factors can discourage the villagers from seeking proper care.	

For peer review only

Supplementary 1 STROBE Statement—Checklist of items that should be included in reports of ***cross-sectional studies***

	Item No	Recommendation	Page No
**Title and abstract**	1	(*a*) Indicate the study's design with a commonly used term in the title or the abstract	1
		(*b*) Provide in the abstract an informative and balanced summary of what was done and what was found	2
**Introduction**			
Background/rationale	2	Explain the scientific background and rationale for the investigation being reported	5-7
Objectives	3	State specific objectives, including any prespecified hypotheses	5-7
**Methods**			
Study design	4	Present key elements of study design early in the paper	7
Setting	5	Describe the setting, locations, and relevant dates, including periods of recruitment, exposure, follow-up, and data collection	7-8
Participants	6	(*a*) Give the eligibility criteria, and the sources and methods of selection of participants	8
Variables	7	Clearly define all outcomes, exposures, predictors, potential confounders, and effect modifiers. Give diagnostic criteria, if applicable	9-10
Data sources/ measurement	8*	For each variable of interest, give sources of data and details of methods of assessment (measurement). Describe comparability of assessment methods if there is more than one group	9-10
Bias	9	Describe any efforts to address potential sources of bias	24
Study size	10	Explain how the study size was arrived at	8
Quantitative variables	11	Explain how quantitative variables were handled in the analyses. If applicable, describe which groupings were chosen and why	9-10
Statistical methods	12	(*a*) Describe all statistical methods, including those used to control for confounding	9-10
		(*b*) Describe any methods used to examine subgroups and interactions	NA
		(*c*) Explain how missing data were addressed	NA
		(*d*) If applicable, describe analytical methods taking account of sampling strategy	NA
		(*e*) Describe any sensitivity analyses	NA
**Results**			
Participants	13*	(a) Report numbers of individuals at each stage of study—eg numbers potentially eligible, examined for eligibility, confirmed eligible, included in the study, completing follow-up, and analysed	12-14
		(b) Give reasons for non-participation at each stage	NA
		(c) Consider use of a flow diagram	NA
Descriptive data	14*	(a) Give characteristics of study participants (eg demographic, clinical, social) and information on exposures and potential confounders	12-14
		(b) Indicate number of participants with missing data for each variable of interest	NA
Outcome data	15*	Report numbers of outcome events or summary measures	12-14

For peer review only - http://bmjopen.bmj.com/site/about/guidelines.xhtml

Main results	16	(*a*) Give unadjusted estimates and, if applicable, confounder-adjusted estimates and their precision (eg, 95% confidence interval). Make clear which confounders were adjusted for and why they were included	14
		(*b*) Report category boundaries when continuous variables were categorized	NA
		(*c*) If relevant, consider translating estimates of relative risk into absolute risk for a meaningful time period	NA
Other analyses	17	Report other analyses done—eg analyses of subgroups and interactions, and sensitivity analyses	NA
**Discussion**			
Key results	18	Summarise key results with reference to study objectives	20
Limitations	19	Discuss limitations of the study, taking into account sources of potential bias or imprecision. Discuss both direction and magnitude of any potential bias	24
Interpretation	20	Give a cautious overall interpretation of results considering objectives, limitations, multiplicity of analyses, results from similar studies, and other relevant evidence	20-24
Generalisability	21	Discuss the generalisability (external validity) of the study results	24
**Other information**			
Funding	22	Give the source of funding and the role of the funders for the present study and, if applicable, for the original study on which the present article is based	25

*Give information separately for exposed and unexposed groups.

Note: An Explanation and Elaboration article discusses each checklist item and gives methodological background and published examples of transparent reporting. The STROBE checklist is best used in conjunction with this article (freely available on the Web sites of PLoS Medicine at http://www.plosmedicine.org/, Annals of Internal Medicine at http://www.annals.org/, and Epidemiology at http://www.epidem.com/). Information on the STROBE Initiative is available at www.strobe-statement.org.

For peer review only

Supplementary 2 Consolidated criteria for reporting qualitative studies (COREQ): 32-item checklist

Developed from:
Tong A, Sainsbury P, Craig J. Consolidated criteria for reporting qualitative research (COREQ): a 32-item checklist for interviews and focus groups. *International Journal for Quality in Health Care*. 2007. Volume 19, Number 6: pp. 349 – 357

**No. Item**	**Guide questions/description**	**Reported on Page #**
**Domain 1: Research team and reflexivity**		
Personal Characteristics		
1. Inter viewer/facilitator	Which author/s conducted the interview or focus group?	10
2. Credentials	What were the researcher's credentials? E.g. PhD, MD	9
3. Occupation	What was their occupation at the time of the study?	9
4. Gender	Was the researcher male or female?	NA
5. Experience and training	What experience or training did the researcher have?	9
Relationship with participants		
6. Relationship established	Was a relationship established prior to study commencement?	24
7. Participant knowledge of the interviewer	What did the participants know about the researcher? e.g. personal goals, reasons for doing the research	8
8. Interviewer characteristics	What characteristics were reported about the inter viewer/facilitator? e.g. Bias, assumptions, reasons and interests in the research topic	NA
**Domain 2: study design**		
Theoretical framework		
9. Methodological orientation and Theory	What methodological orientation was stated to underpin the study? e.g. grounded theory, discourse analysis, ethnography, phenomenology, content analysis	10-11
Participant selection		
10. Sampling	How were participants selected? e.g. purposive, convenience, consecutive, snowball	8-9
11. Method of approach	How were participants approached? e.g. face-to-face, telephone, mail, email	8-9
12. Sample size	How many participants were in the study?	15 (Supplementaries 3-4)
13. Non-participation	How many people refused to participate or dropped out? Reasons?	NA
Setting		
14. Setting of data collection	Where was the data collected? e.g. home,	12

For peer review only - http://bmjopen.bmj.com/site/about/guidelines.xhtml

	clinic, workplace	
15. Presence of non-participants	Was anyone else present besides the participants and researchers?	NA
16. Description of sample	What are the important characteristics of the sample? e.g. demographic data, date	15
Data collection		
17. Interview guide	Were questions, prompts, guides provided by the authors? Was it pilot tested?	11
18. Repeat interviews	Were repeat inter views carried out? If yes, how many?	NA
19. Audio/visual recording	Did the research use audio or visual recording to collect the data?	11
20. Field notes	Were field notes made during and/or after the inter view or focus group?	11
21. Duration	What was the duration of the inter views or focus group?	15
22. Data saturation	Was data saturation discussed?	10
23. Transcripts returned	Were transcripts returned to participants for comment and/or correction?	NA
**Domain 3: analysis and findings**		
Data analysis		
24. Number of data coders	How many data coders coded the data?	11
25. Description of the coding tree	Did authors provide a description of the coding tree?	NA
26. Derivation of themes	Were themes identified in advance or derived from the data?	11
27. Software	What software, if applicable, was used to manage the data?	11
28. Participant checking	Did participants provide feedback on the findings?	NA
Reporting		
29. Quotations presented	Were participant quotations presented to illustrate the themes/findings? Was each quotation identified? e.g. participant number	15-19
30. Data and findings consistent	Was there consistency between the data presented and the findings?	15-19
31. Clarity of major themes	Were major themes clearly presented in the findings?	15-19
32. Clarity of minor themes	Is there a description of diverse cases or discussion of minor themes?	15-19

For peer review only

Supplementary 3 Characteristics of the villagers in the focus group discussions

No.	Village	Content	ID	Unique ID	Participants
1	A	Male FGD	5.59	BM1.1	60s, Retiree
			2.67	BM1.2	40s, Teacher
			2.112	BM1.3	40s, Government servant
			4.113	BM1.4	20s, Soldier
2	A	Male FGD	6.69	BM2.1	50s, Farmer
			5.126	BM2.2	20s, Farmer
			5.129	BM2.3	50s, Farmer
3	A	Female FGD	5.6	BF1.1	30s, Businesswoman
			6.50	BF1.2	50s, Farmer
			2.102	BF1.3	40s, Farmer
4	A	Female FGD	1.122	BF2.1	30s, Farmer
			4.139	BF2.2	40s, Farmer
			3.140	BF2.3	40s, Government servant
5	B	Male FGD	3.61	NM1.1	20s, Government servant
			3.76	NM1.2	30s, Farmer
			4.77	NM1.3	20s, Farmer
6	B	Male FGD	4.87	NM2.1	30s, Farmer
			3.89	NM2.2	30s, Farmer
			1.91	NM2.3	10s, Farmer
7	B	Male FGD	2.93	NM3.1	40s, Farmer
			1.95	NM3.2	30s, Farmer
			1.102	NM3.3	20s, Farmer
8	B	Female FGD	5.41	NF1.1	40s, Farmer
			1.54	NF1.2	40s, Farmer
			2.55	NF1.3	40s, Farmer
9	B	Female FGD	1.98	NF2.1	20s, Farmer
			2.101	NF2.2	20s, Farmer
			3.106	NF2.3	30s, Farmer
10	B	Female FGD	3.99	NF3.1	20s, Farmer
			4.100	NF3.2	40s, Farmer
			2.104	NF3.3	20s, Farmer

For peer review only

For peer review only - http://bmjopen.bmj.com/site/about/guidelines.xhtml

Supplementary 4 Characteristics of the healthcare workers in the in-depth interviews

No.	Village	Content	Unique ID	Age	Sex
1	A	Healthcare worker IDI	BH1	30s	Female
2	A	Healthcare worker IDI	BH2	40s	Female
3	A	Village Health Volunteer IDI	BV1	50s	Male
4	B	Healthcare worker IDI	NH1	30s	Male
5	B	Healthcare worker IDI	NH2	20s	Female
6	B	Healthcare worker IDI	NH3	30s	Female
7	B	Village Health Volunteer IDI	NV1	50s	Male

For peer review only

For peer review only - http://bmjopen.bmj.com/site/about/guidelines.xhtml

Supplementary 5 Themes from the focus group discussions of the villagers

Themes	Quotes, Unique ID
Attributing the cause of malaria to something else other than being bitten by mosquitoes	1. Because your house is not clean. If you drink water with mosquito eggs, you will get fever. *BF 2.3 40s, Government servant*<br>2. When I had malaria, they used '*pee pop*' (ghost) to exorcize it away. *BF 2.1 30s, Farmer*<br>3. Drinking unclean water. *BM1.4 20s, Soldier*<br>4. Drinking unclean water, you will get malaria. *NM 2.3 10s, Farmer*<br>5. Scared of malaria because scared of '*pop hed*' (caused by ghost). *NF1.2 40s, Farmer*
Attributing the cause of malaria to being bitten by mosquitoes	1. Mosquito bite, not sleeping under the bed net, not taking care of oneself. Not protecting oneself. *BF1.1 30s, Businesswoman*<br>2. Caused by mosquitoes. You must be bitten by mosquitoes first. *BF 2.1 30s, Farmer*<br>3. Because of mosquito bites. When you do not sleep under the bed net, the mosquitoes will bite you. *NM1.1 20s, Government servant*<br>4. Do not sleep under the bed net, being bitten by mosquitoes. *NM3.2 30s, Farmer*
Describing an illness using local terms	1. '*Kai luad niao*' (sticky blood fever), when you are dehydrated, your blood will be sticky. When you drink water, your blood will dilute. *BF1.3 40s, Farmer*<br>2. Among men, '*kai luad niao*' (sticky blood fever) is also very common. There were two cases this year, and they died. *BM1.4 20s, Soldier*<br>3. '*Kai nyoong*' (literally translated from Lao as 'mosquito fever', usually referred to malaria) in Katang language is called '*ae muay*'. *NF1.1 40s, Farmer*
Expressing hope to	1. I just hope the health center will be well-equipped with medicines

For peer review only
For peer review only - http://bmjopen.bmj.com/site/about/guidelines.xhtml

improve the health center		and such, and I hope things will get better. I am not criticizing them, I understand that there is not enough budget. *BF1.3 40s, Farmer*
	2.	I hope the health center will be better equipped, with Echo machine, with qualified medical doctors, surgical services. *NM1.2 30s, Farmer*
	3.	I hope the staff will be more knowledgeable on the treatments. *NM3.2 30s, Farmer*
	4.	Improve the health center, I hope they have X-ray machines, proper equipment. *NF1.1 40s, Farmer*
	5.	I hope there will be more medical personnel from other villages stationed here and live here so it will be convenient for us to call on them. *NF1.1 40s, Farmer*
	6.	I hope the health center will be cleaner. There are mosquitoes here at the health center too, so you might even get malaria here! *NF2.2 20s, Farmer*
	7.	I hope the staff at the health center will further their studies and improve their knowledge to help the villagers become healthy. *NM3.1, 40s Farmer*
Expressing hope to improve the district hospital	1.	I hope there will also be a new building at the district hospital. Sometimes there are not enough beds for the patients and the family members have no place to stay. *NM1.1 20s, Government servant; NM1.2 30s, Farmer; NM1.3 20s, Farmer*
Recalling personal experience	1.	We do not have oxygen here in the district hospital. I gave birth to a premature baby once and it died because we did not have oxygen. We were going to the Savannakhet provincial hospital and the baby died on the way in Pakxong. No incubator too. *BF1.1 30s, Businesswoman*
	2.	When I got fever and the health staff gave me the wrong medicine. They do not know. *NM3.3 20s, Farmer*
Expressing opinion	1.	Overall everything is good but only one thing is that medicine is

For peer review only

on the health center	always not enough. *BM1.2 40s, Teacher*<br><br>2. Sometimes, when people are shabbily dressed, the staff do not want to treat them. *BM1.4 20s, Soldier*<br><br>3. If you pay less you get only 'paracetamol'. You won't get well. Last time I had to pay but I felt I recovered fast. Now I pay only 5000 kip I don't get well soon. *NF2.1 20s, Farmer; NF2.2 20s, Farmer; NF2.3 30s, Farmer*
Feeling towards malaria	1. Mosquitoes are scarier than tigers. *NM1.2 30s, Farmer*<br><br>2. Scary! Mosquitoes are scarier than tigers. *NM3.1 40s, Farmer*
Expressing opinion towards malaria elimination	1. Yes, malaria can be eliminated. Destroy the mosquitoes. Spray them. They will be gone. *BF1.3 40s, Farmer*<br><br>2. Elimination is difficult because when people go to the forest, they drink water from the stream. *BF 2.3 40s, Government servant*<br><br>3. It's difficult to eliminate malaria. The only way is to treat. *BM1.4 20s, Soldier*<br><br>4. Cannot be eliminated because our people depend on the forest to make a living, they will just sleep when it gets dark when they are in the forest. *BM2.3 50s, Farmer*<br><br>5. Yes, take care of hygiene, protect oneself when going to the forest. *BM1.4 20s, Soldier*
Expressing difficulties in getting health services	1. '*Kon luai bor dai kau kuk, kon tuk bor dai kau hong mor*' (The rich do not go to jail, the poor do not go to the hospital). *NM1.3 20s, Farmer*<br><br>2. I hope the roads will also be improved. When it is raining, people die because they are not sent to the hospital in time. *NM1.2 30s, Farmer*
Mentioning about the National Health Insurance ("*Kor Por Sor*")	1. I will go to the health center first, because I have to pay only 5000 kip. If I go to a private clinic, I will have to pay at least 50000 kip. *BF 2.1 30s, Farmer*

For peer review only - http://bmjopen.bmj.com/site/about/guidelines.xhtml

Supplementary 6 Themes from the in-depth interviews of the healthcare workers

Themes	Quotes, Unique ID
Mentioning about the National Health Insurance ("*Kor Por Sor*")	1. Now there is the *'Kor Por Sor'* (National Health Insurance) system, where you have to pay only 5000 kip, they will come even when they have common cold. *BH1, 30s Female*<br><br>2. Because of *'Kor Por Sor'* (National Health Insurance), more people come to seek treatment at this health center. *NH1, 30s Male*<br><br>3. Because of *'Kor Por Sor'* (National Health Insurance), we have more patients who come here for treatment. *NH2, 30s Female*
Expressing opinion towards malaria elimination	1. Yes, you can eliminate malaria. You should sleep under the long-lasting insecticide-treated bed nets. In addition, we should also train the village health volunteers to use the rapid diagnostic test (RDT) so we can eliminate malaria. *BH1, 30s Female*<br><br>2. It's difficult to eliminate because people here make a living by going to the mountains, to the forests. We can't stop them from going. They need to make a living. *NH2, 30s Female*<br><br>3. You can't eliminate 100%. People here work in the field, go to the forest, go to the mountains. *NH3, 20s Female*
Describing an illness using local terms	1. I heard the villagers mentioned '*luad niao*', '*luad niao*', is there such thing? *BH2, 40s Female*
Describing the villagers' health-seeking behavior	1. Yes, when they fall sick, they will go to the '*mor pee*' (witch doctor) first. But only for some families. Usually the old. *BV1, 50s Male*<br><br>2. The villagers come to the health center directly as we do not encourage them to go the village health volunteer first. I am afraid that the village health volunteer do not know the right way to treat the patients. *BH2, 40s Female*
Feeling towards the job	1. I am proud of my work. I am a nurse but I also function as a 'doctor' here. I have to work more than what I studied. It is very challenging sometimes but the longer I work, the more experience

For peer review only - http://bmjopen.bmj.com/site/about/guidelines.xhtml

	I have. *NH2, 30s Female*<br><br>2. I am satisfied with my work. Because this work can save lives. *NV1, 50s Male*
Expressing hope to improve the health center	1. The knowledge and capability. I want to learn more. Another thing is the building. Too small. When people are sick they can't sleep here. If possible, I hope for more advanced equipment too. *NH1, 30s Male*<br><br>2. I hope the health center will be expanded. I also want to have machines that can check blood or Echo machine. I also hope that a medical doctor will be stationed here. *NH2, 30s Female*<br><br>3. At this health center the manpower is not enough, we also do not have enough knowledge. I hope people with knowledge will come here to work at this health center. I also hope this health center will be expanded. It is very crowded when there are many sick people and people who give birth. *NH3, 20s Female*
Expressing hope to improve the living condition of the people	1. I hope there will be toilets in every house. *NV1, 50s Male*

For peer review only - http://bmjopen.bmj.com/site/about/guidelines.xhtml
